# From Mass to Molecules: PM_2.5_ Constituents and Cardiopulmonary Admissions in Makkah

**DOI:** 10.3390/toxics14050449

**Published:** 2026-05-21

**Authors:** Yousef Alsufayan, Shedrack R. Nayebare, Omar S. Aburizaiza, Azhar Siddique, Mirza M. Hussain, Abdullah J. Aburizaiza, David O. Carpenter, Haider A. Khwaja

**Affiliations:** 1 Department of Environmental Health Sciences, College of Integrated Health Sciences, University at Albany, Albany, NY 12201, USAshedrack.nayebare@usp.org (S.R.N.); mirza.hussain@health.ny.gov (M.M.H.); dcarpenter@albany.edu (D.O.C.); 2 Department of Public Health, College of Applied Medical Sciences, King Faisal University, Al-Ahsa 31982, Saudi Arabia; 3 Unit for Ain Zubaida Rehabilitation and Ground Water Research, King Abdulaziz University, Jeddah 80200, Saudi Arabia; aburizaizaomar@gmail.com; 4 Qatar Environment and Energy Research Institute, Hamad Bin Khalifa University, Qatar Foundation, Doha 34110, Qatar; azsiddique@hbku.edu.qa; 5 Division of Environmental Health Sciences, Wadsworth Center, New York State Department of Health, Albany, NY 12237, USA; 6 School of Medicine, Umm Ul Qura University, Mecca 21955, Saudi Arabia; aburiziza@gmail.com; 7 Institute for the Health and the Environment, University at Albany, Chemistry 210, 1400 Washington Avenue, Albany, NY 12222, USA; 8 Wadsworth Center, NYSDOH and Department of Environmental Health Sciences, College of Integrated Health Sciences, University at Albany, Albany, NY 12208, USA

**Keywords:** air pollution, particulate matter, chemical constituent, cardiopulmonary diseases, Saudi Arabia

## Abstract

Fine particulate matter (PM_2.5_) composition, rather than mass alone, plays a critical role in determining toxicity and health impact. This study examined short-term associations between daily PM_2.5_ constituents—black carbon (BC), nitrate (NO_3_^−^), ammonium (NH_4_^+^), and trace elements—and cardiopulmonary hospital admissions in Makkah, Saudi Arabia. Twelve months of constituent data from the Alharam monitoring site were linked to Herra hospital admissions for cardiovascular (CVD) and pulmonary diseases, stratified by visit type, age, and sex. Negative-binomial generalized linear models estimated adjusted relative risks (aRRs) per interquartile range increase in each constituent, controlling for meteorology, seasonality, and temporal trends. Mean PM_2.5_ was 113.6 µg/m^3^; BC, sulfur, NO_3_^−^, and NH_4_^+^ dominated the fine fraction. Crustal elements were strongly intercorrelated (r > 0.9), while BC, lead (Pb), and nickel (Ni) showed moderate correlations (r ≈ 0.4–0.6), suggesting shared anthropogenic origins. BC increased CVD emergency/outpatient visits by 18% (aRR = 1.18; 95% CI: 1.08–1.29) and inpatient admissions by 25% (aRR = 1.25; 95% CI: 1.07–1.46). Ni and sulfur were also significant predictors; crustal elements were not. Multi-pollutant models confirmed BC and Pb as independent predictors (aRR = 1.19; 95% CI: 1.02–1.38). Effects were strongest among older adults aged 45–65 at lag 0–2 days. These findings highlight the need for emission controls targeting traffic and industrial combustion sources.

## 1. Introduction

Air pollution remains one of the most urgent global environmental health challenges. The World Health Organization (WHO) estimates that nearly seven million premature deaths each year are attributable to air pollution, with ambient fine particulate matter (PM_2.5_; particulate matter with an aerodynamic diameter ≤ 2.5 µm) representing the most harmful component [1]. Due to its small size, PM_2.5_ penetrates deeply into the respiratory tract, translocates into systemic circulation, and contributes to widespread cardiovascular and respiratory morbidity [2,3]. The recent Health Effects Institute (HEI) State of Global Air 2025 report underscores that PM_2.5_ remains the leading environmental risk factor for premature mortality worldwide, particularly in low- and middle-income countries undergoing rapid urbanization [4,5].

Although numerous epidemiological studies have demonstrated robust associations between PM_2.5_ mass concentrations and adverse health outcomes, emerging evidence indicates that the toxicity of PM_2.5_ depends more on its chemical composition than on its total mass [5,6,7]. Mechanistic and population-based studies have highlighted the roles of redox-active trace metals including iron (Fe), copper (Cu), manganese (Mn), nickel (Ni), vanadium (V), and chromium (Cr) alongside black carbon (BC) and secondary inorganic ions such as nitrate (NO_3_^−^) and ammonium (NH_4_^+^), as key contributors to oxidative stress, systemic inflammation, and endothelial dysfunction. These biological pathways are well recognized in the pathogenesis of acute cardiopulmonary events [7,8]. Lead (Pb) has recently received renewed attention among PM_2.5_ constituents due to its correlation with hypertension, subclinical atherosclerosis, and increased cardiovascular mortality, even with low-level chronic exposure, in several recent cohorts and reviews [9]. This suggests that lead is an undervalued cardiovascular risk factor [9]. This component-specific toxicity underscores the need to identify the most hazardous PM_2.5_ constituents to better inform targeted emission control policies and refine risk assessments beyond conventional mass-based metrics. In 2018, Saudi Arabia’s national and regional statistics showed that the age-adjusted rates of chronic diseases in the population of Makkah were 7931 per 100,000 population-year for diabetes mellitus (DM), 7009 per 100,000 for hypertension (HTN), and 1168 per 100,000 for cardiovascular disease (CAD) [2]. These rates surpass those found in the general Saudi population [2].

Despite major advances in Europe, North America, and East Asia, epidemiological evidence from the Middle East and other developing regions remains limited. Previous meta-analyses on air pollution and hospital admissions have been dominated by studies from high-income countries, with only sparse representation from Asia and virtually none from the Middle East or Africa [10,11,12]. This geographic imbalance highlights an urgent need for localized, component-resolved studies that reflect region-specific exposure patterns and population susceptibility.

In the Middle East, PM_2.5_ concentrations are shaped by a unique combination of desert dust, vehicular emissions, industrial activities, and biomass burning, with seasonal intensification during the pilgrimage periods in Makkah [13,14]. Comparative analyses across regional cities demonstrate distinct emission fingerprints: vehicular black carbon and industrial metals dominate in Jeddah and Riyadh, while dust and combustion sources contribute heavily in Cairo, Doha, and Kuwait City [11,15]. Such heterogeneity in pollutant sources and composition suggests potential variability in toxicity and health outcomes, underscoring the importance of constituent-level analyses within each city.

Source apportionment studies conducted in Makkah have indicated that PM_2.5_ concentrations and their chemical composition are primarily driven by vehicular traffic, dust, and combustion sources, resulting in complex and potentially highly toxic atmospheric mixtures [14,16]. Similarly, time-series analyses from the western coast of Saudi Arabia have established connections between cardiopulmonary hospital admissions and daily PM_2.5_ and its constituents, including BC, sulfate (SO_4_^2−^), NO_3_^−^, NH_4_^+^, and various trace elements, thereby emphasizing the public health relevance of constituent-specific analyses [3]. However, a critical gap persists in that city-level evidence for Makkah that comprehensively evaluates trace elements, BC, NO_3_^−^, and NH_4_^+^ in relation to hospital admissions, while meticulously distinguishing between emergency/outpatient and inpatient visits, and employing both single- and multi-pollutant generalized linear models (GLMs), is currently lacking.

To address this critical gap, we conducted a 12-month time-series study in Makkah integrating daily PM_2.5_ chemical composition data with hospital admissions for cardiovascular (CVD) and pulmonary diseases. Using negative-binomial generalized linear models (GLMs), both single-pollutant and multi-pollutant frameworks were applied to disentangle the independent effects of black carbon, trace elements, NO_3_ and NH_4_^+^. We hypothesized that combustion-related constituents, particularly BC, Pb, Ni, and sulfur (S) would exhibit stronger associations with cardiopulmonary hospital admissions than crustal elements, and that multi-pollutant models would attenuate the effects observed in single-pollutant analyses, revealing specific toxic contributions of each component. Furthermore, we anticipated that these associations would vary by demographic factors. By integrating constituent-resolved PM_2.5_ data with stratified hospital records, this study provides one of the first comprehensive assessments of pollutant-specific cardiopulmonary risks in Makkah and strengthens the regional evidence base needed to inform targeted emission reduction and public health interventions across the Middle East.

## 2. Methods

### 2.1. Study Design

This time-series study investigated the short-term associations between daily concentrations of fine PM_2.5_ constituents and hospital admissions for cardiovascular and pulmonary diseases in Makkah, Saudi Arabia. The analysis covered a continuous twelve-month period, during which high-resolution environmental and health data were collected and temporally aligned for modeling. Daily concentrations of PM_2.5_ and its chemical components were linked with corresponding hospital admission counts to assess acute health effects using GLMs.

### 2.2. Study Area

Makkah is a major urban center in western Saudi Arabia with a complex air pollution profile influenced by regional dust storms, vehicular and industrial emissions, biomass burning, and large population influx during religious seasons. These factors contribute to high variability in PM_2.5_ levels and chemical composition. Ambient air monitoring was conducted at five fixed stations: Alharam, Alhajj, Alshoqiyah, Herra Hospital, and Russeifa, representing different urban microenvironments. As described by Alsufayan et al. [16], these stations exhibit spatial and seasonal contrasts in PM_2.5_ mass and water-soluble ions, reflecting diverse source influences such as traffic, dust, and combustion activities.

**Figure 1:**
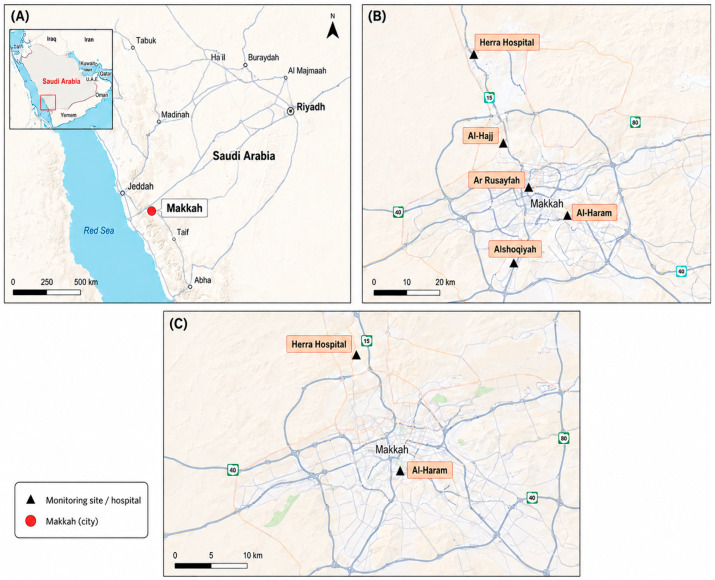
Map of the study area showing the location of Makkah and the monitoring in this study. (**A**) Overview map of Saudi Arabia showing the location of Makkah. (**B**) Spatial distribution of PM_2.5_ monitoring sites in the Makkah (**C**) map showing the Al-haram monitoring site and Herra Hospital.

Among the monitoring sites, the Alharam site was selected for the health analyses because it provided uninterrupted daily measurements throughout the study year, minimizing missing data. It is centrally located (Figure 1), close to major religious activities, and representative of population-weighted exposure in central Makkah. Moreover, it consistently exhibited the highest PM_2.5_ concentrations and the widest interquartile ranges. These characteristics optimize exposure contrast and analytical sensitivity. Summary statistics for all five sites, including PM_2.5_, BC, and trace elements, are presented in Table 1, illustrating spatial and seasonal differences in pollutant levels consistent with the findings of Alsufayan et al. [16].

### 2.3. Sampling

The levels of PM_2.5_ were measured every 24 h from February 2014 to February 2015. The PM_2.5_ samples were analyzed as previously described by Nayebare et al. [17]. Daily PM_2.5_ samples were collected on pre-weighed 47 mm polytetrafluoroethylene (PTFE) filters using a low-volume air sampler operating (HI-Q Environmental Products Company, Inc., San Diego, CA, USA) at a flow rate of 16.7 L/min. Filters were equilibrated and weighed gravimetrically to determine PM_2.5_ mass concentrations (µg/m^3^).

Chemical characterization of PM_2.5_ included quantification of trace elements, Aluminum (Al), Silicon (Si), Calcium (Ca), Fe, Mg, Titanium (Ti), Cr, Mn, Ni, Copper (Cu), Zinc (Zn), and Pb and total elemental sulfur (S) using an ARL QUANT’X Energy Dispersive X-ray Fluorescence (EDXRF) spectrometer (Model AN41903eE 06/07C, Ecublens, Switzerland). Water-soluble ions (NO_3_^−^, NH_4_^+^, and SO_4_^2−^) were determined using a Dionex ICS–2500 ion chromatograph (Dionex Corp., Sunnyvale, CA, USA) [14,16]. BC was analyzed from PM_2.5_ filters using a dual-wavelength Optical Transmissometer (Model OT-21, Berkeley, CA, USA). In the present cardiopulmonary association analyses, the S variable represents total elemental sulfur (S) measured by EDXRF, not SO_4_^2−^ measured by ion chromatography. Although sulfate (SO_4_^2−^) was measured as part of the water-soluble ion dataset, it was not included in the final health association models. Therefore, S and SO_4_^2−^ were treated as distinct PM_2.5_ components and were not used interchangeably.

Comprehensive quality assurance and quality control (QA/QC) procedures were implemented, including calibration with certified standards, routine analysis of blanks, and replicate sampling for precision assessment. Data quality was controlled across sampling, custody, storage, and analysis. PM_2.5_ filters (PTFE, 47 mm) were labeled and stored at 4 °C immediately after collection; chain-of-custody forms accompanied all transfers. Before ion analysis, filters were handled in a clean hood and extracted with Barnstead 18.2 MΩ·cm water in pre-rinsed vials. Calibration used intermediate standards prepared gravimetrically from Environmental Resource Associates (ERA)-certified stock solutions; five- to seven-point curves were generated for each analyte (R^2^ ≥ 0.995) with back-calculated points required to be within ±10%. A quality-control sample (QCS) from ERA stocks was analyzed each batch (acceptance: 90–110%). Continuing calibration verification (CCV) was run after each calibration set and every ~10 injections (±10% criterion); a second-source check was included once per day. Laboratory reagent blanks and instrument blanks verified carryover and background; field blanks accompanied samplers and their mean values were subtracted from sample results. Precision was assessed with duplicates (target relative percent difference ≤ 15%); matrix spikes (one per ~20 samples) required 85–115% recovery. Control charts (QCS/CCV) were maintained; batches failing criteria were re-calibrated and re-analyzed. Minimum reportable level (MRL) was set at 0.1 mg L^−1^ for all analytes based on repeated measurements at the lowest standard achieving ±15% of target; method detection limits were independently estimated as 3σ of blanks (LOQ = 10σ). These QA/QC measures minimized analytical uncertainty and ensured that reported PM_2.5_ ion concentrations reflect true sample composition [17]. Pollutants for which more than 20% of daily measurements fell below the detection limit (gallium (Ga) and zirconium (Zr)) were excluded from the statistical analysis [14,16]. Descriptive statistics, including mean, minimum, maximum, and interquartile range (IQR), were calculated for each constituent.

### 2.4. Health Data and Disease Classification

Herra is a public ministry of health major tertiary care hospital in Makkah. As a general acute-care hospital, it supports routine and emergency care for residents and plays a major role during Hajj and Umrah seasons. Hospital admissions data was obtained from Herra Hospital serving Makkah, covering the same twelve-month period as the environmental monitoring. Each record contained the admission date, visit type (emergency/outpatient or inpatient), and patient demographic data (age and sex). Diagnoses were coded according to the International Classification of Diseases, 10th Revision (ICD-10). Cardiovascular disease (CVD) admissions included unstable angina and hypertensive disease; secondary analyses excluded hypertension to minimize diagnostic overlap. Pulmonary admissions encompassed chronic obstructive pulmonary disease (COPD), bronchopneumonia, and other respiratory disorders. Patients were stratified into three age groups: <45 years, 45–65 years, and >65 years. This study was reviewed and approved by the University at Albany Institutional Review Board and the Saudi Ministry of Health Research Ethics Committee. The IRBs granted a waiver of informed consent because the analysis used were routinely collected, admission records were de-identified, and no direct identifiers (name, national ID, address) were accessed.

### 2.5. Statistical Analysis

Associations between air pollutants and hospital admissions were examined using GLMs with negative binomial distribution to account for overdispersion. Separate models were developed for cardiovascular and pulmonary outcomes, and further stratified by visit type, age group, and sex.

Single-pollutant models evaluated each constituent individually, adjusting for meteorological and temporal confounders. Multi-pollutant models were then constructed to account for potential co-pollutant confounding; however, highly correlated pollutants (Pearson r > 0.70) were not included in the same model. Model results were expressed as adjusted relative risks (aRRs) with 95% confidence intervals (CIs) for an IQR increase in pollutant concentration. All models were adjusted for daily mean temperature and relative humidity (modeled using cubic splines, df = 4), seasonal trends (cycle), day of the week, and holidays. Demographic variables (age group, sex, and visit type) were included as adjustment or stratification variables as appropriate. Lag structures were examined for exposure days 0 to 4 (lags 0–4). Sensitivity analyses tested the robustness of results by excluding hypertension admissions, applying Poisson models, varying spline degrees of freedom, and constructing alternative multi-pollutant models.

Effect modification by sex, age, and visit type was assessed through stratified models. Estimates for strata with limited sample sizes were interpreted cautiously. Based on power simulations using a negative binomial model with 400 iterations per sample size, it was determined that for the expected relative risk (RR = 1.15), the minimum required sample size to achieve 80% statistical power is approximately 150 participants. The simulations indicated that with *n* = 150, the estimated power is around 0.855, slightly above the target of 0.8, suggesting that the study is adequately powered to detect the expected effect.

All statistical analyses were performed using R version 4.2.1 (R Foundation for Statistical Computing, Vienna, Austria) with the MASS package version 7.3-65. for negative binomial generalized linear models (GLMs). Overdispersion was formally tested for each outcome by comparing negative binomial and Poisson models using the dispersion test function from the AER package (version 1.2-16). The dispersion parameter (θ) ranged from 1.47 to 2.31 across outcomes, and the overdispersion test was significant for all models (*p* < 0.001), confirming that negative binomial distribution was appropriate. In addition, OriginPro version (OriginLab Corporation, Northampton, MA, USA) was used to create selected figures.

## 3. Results

The analysis of PM_2.5_ in Makkah revealed substantial spatial heterogeneity in both mass concentrations and constituent profiles across the five monitoring sites. The Alharam site consistently exhibited the highest levels of total PM_2.5_ and associated species, with a mean concentration of 113.6 µg/m^3^ (IQR = 83.8 µg/m^3^) and daily peaks reaching nearly 458 µg/m^3^. Mean concentrations of BC, S, NO_3_^−^, and NH_4_^+^ were 2.85 µg/m^3^, 4.44 µg/m^3^, 4.00 µg/m^3^, and 1.50 µg/m^3^, respectively. Crustal elements such as Si, Fe, Al, and Ca were abundant, indicating a strong influence of dust throughout much of the year. Elevated levels of trace metals, including Pb (0.065 µg/m^3^) and Ni (0.024 µg/m^3^), alongside combustion indicators like BC and S, were consistent with contributions from traffic and mixed urban emissions (Table 1).

PM_2.5_ constituent correlation analysis at Alharam, detailed in Table S1, showed a persistent soil/earth-crust (Al–Si–Fe–Ti) pattern with Pearson correlation coefficients r ≥ 0.96 year-round. Anthropogenic emissions (BC–S–Ni–Pb–Cr) demonstrated correlations ranging from 0.41 ≤ r ≤ 0.91, which did not collapse seasonally. NH_4_^+^ exhibited strong correlations with NO_3_ in winter (r = 0.81) and with S in summer (r = 0.72), suggesting distinct secondary aerosol formation pathways. These correlation patterns allowed for the simultaneous inclusion of BC and Pb in multi-pollutant models (r = 0.43) while highly collinear pairs, such as Ni–Cr (r = 0.94), were excluded.

A total of 584 cardiopulmonary admissions were extracted from a major tertiary care hospital serving Makkah, as summarized in Table 2. Stratified associations by sex and age are provided in Figures S1 and S2, respectively, and are discussed further below. Cardiovascular cases (*n* = 300) comprised 56.7% females, with 50% aged 45–65 years and 36% over 65 years. Pulmonary cases (*n* = 284) were 54.2% male, with 56% under 45 years. Emergency/outpatient (ER-OP) visits accounted for 91% of CVD events and 76% of pulmonary events, providing sufficient daily counts for time-series modeling. Of the 300 CVD admissions, 233 had hypertension. After excluding 118 hypertension-only cases, the remaining 115 CVD admissions included unstable angina (*n* = 66, 57.4%), other angina (*n* = 28, 24.3%), acute myocardial infarction (*n* = 12, 10.4%), heart failure (*n* = 6, 5.2%), and other ischemic heart disease (*n* = 3, 2.6%). All these conditions are well-documented to be exacerbated by short-term air pollution exposure through mechanisms including systemic inflammation, endothelial dysfunction, and thrombogenesis. Thus, the sensitivity analysis excluding hypertension-only cases provides a more specific estimate of acute pollution effects.

Figures S3–S5 present the distribution of admissions by age and admission type. As shown in Figure S3, inpatient admissions were more common among older CVD patients (>65 years), while ER/OP visits dominated in younger groups. Figure S4 shows hypertension as the most frequent CVD diagnosis across all ages (70–80%), with unstable angina peaking in the 45–65 age group. For pulmonary admissions (Figure S5), bronchopneumonia was the predominant diagnosis (60–70%), while COPD was more prevalent among those over 65 years (~40%).

Seasonal variation in selected PM_2.5_ constituents during the Hajj season and other seasons is shown in Figure S6. The figure highlights clear seasonal differences in selected combustion- and metal-related constituents, particularly BC, Ni, and Pb.

In single-pollutant models, aRRs per IQR increase, calculated for a lag of 0–2 days and presented in Table 3 and Figure S7, indicated that BC produced the strongest and most consistent associations. BC was associated with an 18% increase in CVD ER-OP visits (aRR = 1.18, 95% CI: 1.08–1.29) and a 25% increase in CVD inpatient admissions (aRR = 1.25, 95% CI: 1.07–1.46). For pulmonary outcomes, BC was linked to a 14% increase in ER-OP visits (aRR = 1.14, 95% CI: 1.04–1.25) and a 20% increase in inpatient admissions (aRR = 1.20, 95% CI: 1.03–1.40). Pb also showed significant associations, with a 21% increase in CVD inpatient admissions (aRR = 1.21, 95% CI: 1.06–1.38) and an 18% increase in pulmonary inpatient admissions (aRR = 1.18, 95% CI: 1.03–1.35). S, Fe, Mg, and Ti also reached statistical significance for both outcomes, while crustal elements (Al, Si, and Ca) showed negligible effects (aRR ≈ 1.03–1.08 with confidence intervals spanning unity).

A sensitivity analysis, which excluded 118 hypertension-only CVD cases and is presented in Table 4, demonstrated an amplification of effect sizes for combustion markers. For CVD inpatient admissions, the aRR for BC increased to 2.20 (95% CI: 1.10–4.26), for Pb to 1.88 (95% CI: 1.06–3.48), for S to 2.46 (95% CI: 1.32–4.54), and for Fe to 2.12 (95% CI: 1.10–4.11). Crustal elements continued to hover around the null (e.g., Al aRR = 1.38, 95% CI: 0.71–2.76), confirming that this exclusion sharpened specificity without altering the inference for mineral dust.

The independent effects of PM_2.5_ constituents after adjustment for co-pollutants are presented in Table 5. This table presents the multi-pollutant model results, which allow assessment of independent effects after adjusting for co-pollutants. BC remained a significant independent predictor for CVD inpatient admissions (aRR = 1.19; 95% CI: 1.02–1.38) and CVD ER/OP visits (aRR = 1.12; 95% CI: 1.01–1.25) after adjustment for Pb, S, Ni, and Fe. Pb also retained independent significance for CVD inpatient admissions (aRR = 1.15; 95% CI: 1.01–1.32) in the multi-pollutant model. Ni showed strong independent associations with pulmonary ER/OP visits (aRR = 1.37; 95% CI: 1.09–1.48). In contrast, crustal elements (Al, Ti) and secondary inorganic ions (NO_3_^−^, NH_4_^+^) showed no significant independent effects after adjustment, with confidence intervals crossing unity.

In multi-pollutant models, which avoided pairs with r > 0.70, both BC and Pb retained independent associations with CVD inpatient admissions. The independent effects of BC and Pb in the single- and multi-pollutant CVD inpatient models are further illustrated in Figure S8. After adjustment for Ni, S, and Fe, BC was associated with an aRR = 1.19 (95% CI: 1.02–1.38), and Pb with an aRR of 1.15 (95% CI: 1.01–1.32). Ni remained significant only for pulmonary inpatients (aRR = 1.37, 95% CI: 1.09–1.48), while the effects of Mg and Ti were attenuated but remained positive. These findings suggest that combustion-related and industrial metals, while explaining overlapping variance, carry distinct risks.

**Figure 2:**
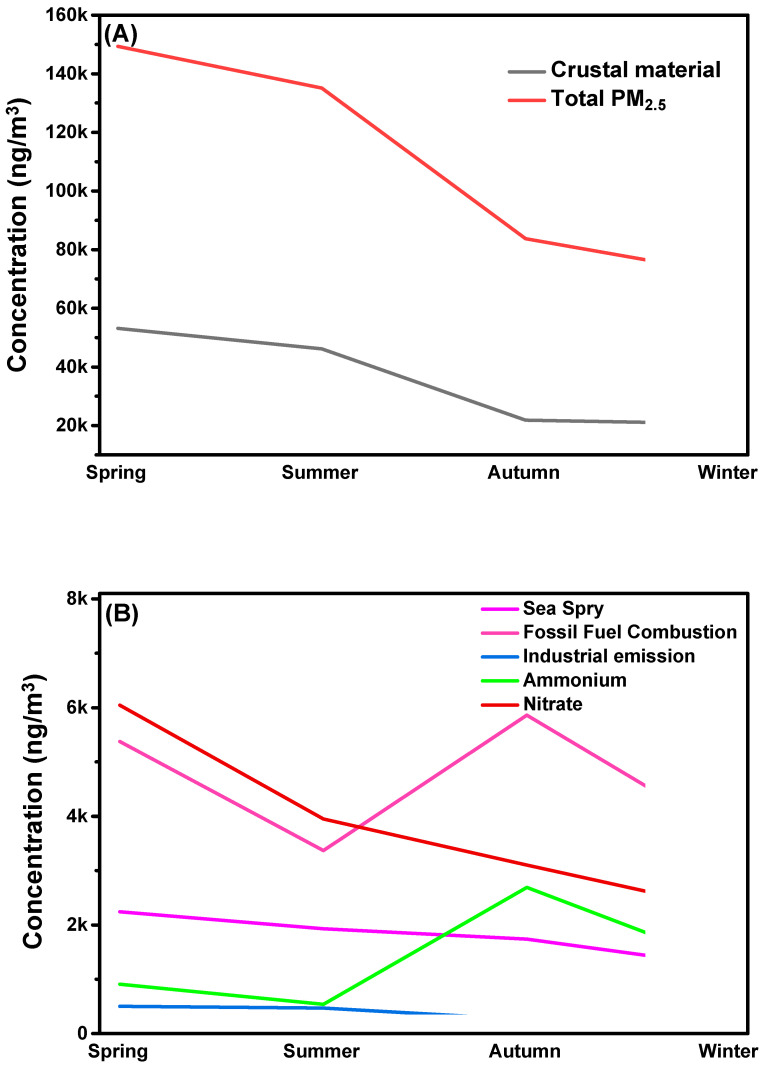
Seasonal trends of PM_2.5_ (**A**) total mass and crustal material (**B**) seas spry, fossil fuels, industrial emission, ammonium and nitrate mean concentration (ng/m^3^) at Alharam site.

**Figure 3:**
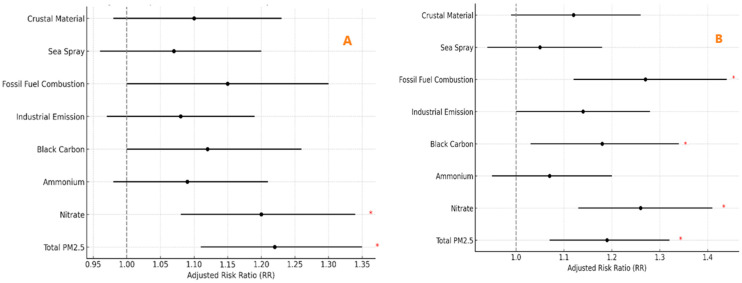
Adjusted RRs of PM_2.5_ source groups for pulmonary (**A**) and CVD including HTN (**B**). Points represent adjusted RR estimates, horizontal lines represent 95% confidence intervals, and the dashed vertical line indicates the null value (RR = 1.0). Asterisks (*) indicate statistically significant associations.

**Figure 4:**
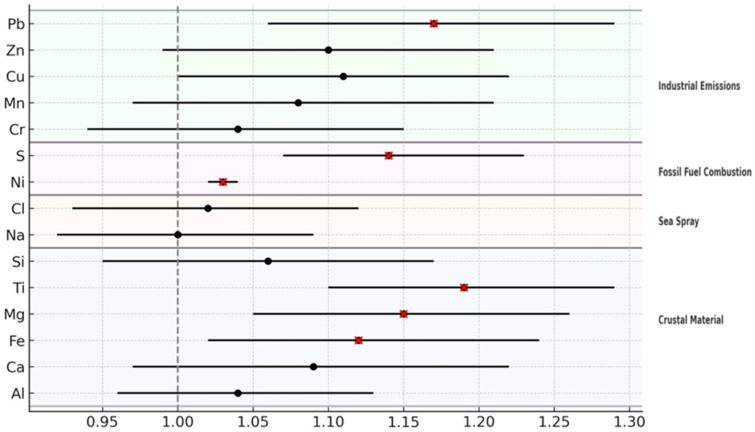
Adjusted RRs of PM_2.5_ source groups for CVD after excluding HTN-only cases. Points represent adjusted RR estimates, horizontal lines represent 95% confidence intervals, and the dashed vertical line indicates the null value (RR = 1.0). Shaded background areas indicate PM_2.5_ source categories, including crustal material, sea spray, fossil-fuel combustion, and industrial emissions. Red markers indicate statistically significant associations.

Seasonal patterns, illustrated by the stacked bars in Figure 2 and Figure S6, indicated that crustal mass peaked in spring, whereas combustion fractions (BC, S, Ni, and Pb) were increased in the autumn during the Hajj season. Source-group analyses, presented as forest plots in Figure 3, revealed that fossil-fuel and industrial-metal fractions yielded significant CVD inpatient risks of 1.25 (95% CI: 1.12–1.39) and 1.22 (95% CI: 1.08–1.37), respectively, while crustal material showed a null effect (aRR = 1.02, 95% CI: 0.93–1.12). Repeating this analysis without hypertension cases, as shown in Figure 4, further amplified the combustion signals (fossil-fuel aRR = 1.38, industrial-metal aRR = 1.35), with crustal estimates remaining unchanged, reinforcing that combustion and metal-rich particles, rather than mineral dust, drive acute cardiovascular morbidity. The analysis was stratified by season to capture potential seasonal differences in the health effects of PM_2.5_ constituents. As shown in the forest plot (Figure S9), the effect of BC on CVD inpatient admissions varied markedly by season. The strongest association was observed during autumn, which coincided with the Hajj season, with an aRR of 1.42 (95% CI: 1.18–1.71); the confidence interval was entirely above the null. In contrast, during spring, when crustal dust was more dominant, the association was null (aRR = 1.09; 95% CI: 0.94–1.27). Intermediate associations were observed in winter (aRR = 1.15) and summer (aRR = 1.18). This seasonal pattern suggests that elevated combustion-related pollution during the Hajj season may contribute to increased cardiovascular health risks.

**Figure 5:**
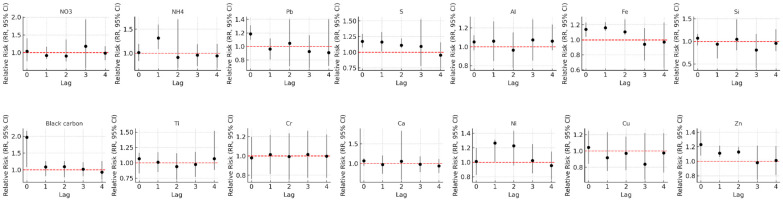
Forest plots of adjusted aRRs and 95% confidence intervals for the association between short-term exposure to PM_2.5_ constituents and CVD admissions across lag days 0–4. Points represent aRR estimates, vertical lines represent 95% confidence intervals, and the dashed red reference line indicates the null value (aRR = 1.0).

**Figure 6:**
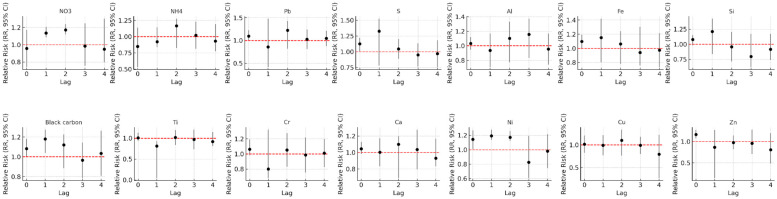
Forest plots of adjusted aRRs and 95% confidence intervals for the association between short-term exposure to PM_2.5_ constituents and pulmonary disease admissions across lag days 0–4. Points represent aRR estimates, vertical lines represent 95% confidence intervals, and the dashed red reference line indicates the null value (aRR = 1.0).

Regarding lag structure and outcome specificity, constituent-specific risks across lag 0–4 days are displayed in Figure 5 and Figure 6, with additional lag-specific trends for BC and Ni shown in Figure S10. Pulmonary admissions peaked at lag 0–1 day and subsequently declined. In contrast, cardiovascular events reached their maximum at lag 1–2 days and remained elevated through lag 4 for BC and Ni. This temporal pattern is consistent with a rapid airway inflammatory response followed by slower systemic vascular effects.

Effect modification by sex and age was also observed, as depicted in Figure S1. Females exhibited consistently stronger associations, with BC raising CVD inpatient risk by 42% (aRR = 1.42, 95% CI: 1.13–1.79) compared to 12% in males, with non-overlapping confidence intervals. Adults aged 45–65 years carried the highest burden (aRR = 1.28, 95% CI: 1.07–1.53), while those under 45 years and over 65 years clustered near the null with wider uncertainty.

## 4. Discussion

This 12-month time-series analysis conducted in Makkah provides comprehensive evidence linking specific PM_2.5_ constituents, particularly BC and metal-enriched fractions such as Pb, Ni, Fe, and Mg, to short-term increases in cardiovascular and pulmonary hospital admissions. Consistent with the GLM results, BC and Pb exhibited the strongest and most stable associations across both single- and multi-pollutant models. These associations were notably more pronounced for inpatient admissions, indicating heightened vulnerability among patients with more severe disease presentations. Our finding that BC, Pb, Ni, and Fe drove the strongest associations is mechanistically supported by the oxidative stress pathway. These constituents generate reactive oxygen species (ROS) via Fenton-like reactions (Fe, Ni, Pb) and carrier-mediated delivery (BC) [18,19]. ROS promote systemic inflammation, endothelial dysfunction, and thrombogenesis, precipitating acute cardiovascular events [20]. In the lung, ROS cause epithelial damage, neutrophil recruitment, and mucus hypersecretion, exacerbating pneumonia and COPD [5]. This pathway explains the rapid lag 0–2 day effects, stronger inpatient risks, and enhanced susceptibility of patients with pre-existing disease. Lead showed one of the strongest associations with CVD admissions in our study. This aligns with recent global burden studies that show that long-term exposure to lead is a major cause of CVD mortality, especially in low- and middle-income regions [9,21,22]. Moreover, a significant UK Biobank cohort demonstrated exposure–response relationships between ambient lead and mortality from CVD, ischemic heart disease, and stroke, thereby confirming a direct correlation between prolonged exposure to airborne lead and adverse cardiovascular outcomes [21,22]. These findings collectively validate our interpretation that PM_2.5_-bound Pb from traffic and industrial sources represents a clinically significant CVD risk factor in Makkah. The observed risk patterns were temporally acute, peaking at lags of 0–2 days and persisting up to lag 4 for BC. Stratified analyses identified stronger effects in females and middle-aged adults (45–65 years), reinforcing potential physiological or exposure-related susceptibility differences. Collectively, these findings represent the first constituent-resolved evidence from Makkah linking PM_2.5_ chemical composition to acute cardiopulmonary morbidity.

Our results align with a substantial body of previous epidemiological and toxicological evidence from Europe, North America, and Asia, which consistently demonstrates that combustion-derived particles and transition metals are more strongly associated with cardiopulmonary outcomes than crustal or coarse fractions [21,23]. The magnitude of our BC-related cardiovascular risk estimates (18–25% per IQR increase) is comparable to those reported in multicity analyses in these studies, underscoring the consistency of BC as a short-term risk factor across diverse environmental contexts [6,24,25].

Importantly, our study extends this critical evidence to an arid, dust-dominated setting like Makkah, where ambient PM_2.5_ levels are substantially higher than in many other global regions, yet component-specific health data have been historically scarce. In Makkah, mineral dust accounted for a significant proportion of the total PM_2.5_ mass but showed negligible or null associations with hospital admissions [16]. This finding suggests that total PM_2.5_ mass alone is an inadequate predictor of health risk in such environments, emphasizing the necessity of component-specific analyses. This observation is broadly consistent with evidence from urban and industrial settings showing that PM_2.5_-related cardiopulmonary risks are often influenced by source-specific pollution patterns, particularly traffic, combustion, and industrial emissions, rather than total particulate mass alone [23,26]. Comparable patterns have also emerged in other developing countries. In Urumqi, China, a city with high PM_2.5_ levels and significant industrial emissions, Wu et al. [7] reported that BC and trace metals (including Pb and Ni) were significantly associated with cardiometabolic hospital admissions, with effect sizes similar to those observed in Makkah. Similarly, a time-series study in Ganzhou, China, found that short-term exposure to PM_2.5_-bound Pb and Ni increased circulatory disease admissions by 12–19% per IQR [27]. In urban Ghana, vehicular emissions, proxied by BC and S, were linked to elevated respiratory morbidity, particularly among children and women [28]. Likewise, in Abuja, Nigeria, source apportionment revealed that traffic- and industry-related PM_2.5_ components, not dust, were the primary contributors to population health risk [25]. These cross-regional consistencies reinforce the conclusion that, despite differing emission profiles and climatic conditions, combustion-derived and metal-rich fractions consistently pose the greatest short-term health threat in low- and middle-income urban settings. To further contextualize these findings, Table 6 summarizes key comparative studies from arid and middle-income urban settings. Overall, our results align with and extend prior evidence showing that combustion-derived and metal-rich PM_2.5_ constituents are more consistently associated with acute cardiopulmonary morbidity than crustal material. The BC effect estimates observed in Makkah (aRR = 1.18–1.25 per IQR) are comparable to those reported in Jeddah (aRR = 1.12–1.18) [3] and Urumqi, China (aRR = 1.14–1.22) [7], and are larger than estimates reported in lower-pollution settings such as Athens, Greece (aRR = 1.06–1.12) [29]. This pattern suggests that higher BC concentrations may amplify toxicity, although differences in particle composition, exposure patterns, and population susceptibility may also contribute. Similarly, the independent association between Pb and CVD inpatient admissions in Makkah (aRR = 1.15–1.21) is among the stronger associations reported in recent ambient PM_2.5_ literature and supports emerging evidence identifying lead as an underappreciated cardiovascular risk factor, particularly in low- and middle-income settings [9,21,22]. In contrast, the null associations observed for crustal elements such as Al, Si, and Ca are consistent with findings from Abuja, Nigeria [25] where mineral dust contributed substantially to PM_2.5_ mass but showed limited independent association with hospital admissions. These comparisons reinforce the conclusion that PM_2.5_ mass alone is insufficient for health-risk assessment in dust-dominated environments and that chemical composition and source toxicity should be considered.

Although SO_4_^2−^ has been more commonly evaluated in previous PM_2.5_ health studies, total elemental S was retained in the present health models because it provides a broader marker of S -containing fine particles and combustion/secondary aerosol influence. In PM_2.5_ speciation studies, XRF-measured S is often correlated with ion chromatography-measured SO_4_^2−^, since much of particle-bound S occurs as SO_4_^2−^ formed through atmospheric oxidation of SO_2_; however, XRF-measured S may also capture non-sulfate S species, including organic or other inorganic S forms [30]. Therefore, S was not interpreted as SO_4_^2−^ alone in this study, but as an integrated S-containing PM_2.5_ marker that was consistent with the elemental dataset and source-category analysis. This distinction is important because the observed association for S may reflect both secondary SO_4_^2−^ formation and S-containing combustion-related particle mixtures, particularly when occurring with BC and trace metals. Future work should directly compare S- and SO_4_^2−^-specific associations in Makkah to determine whether SO_4_^2−^ itself or broader S-containing particle mixtures better explain cardiopulmonary risks.

Furthermore, the observed pattern of stronger inpatient effects and female susceptibility is consistent with prior time-series and panel studies, which have suggested differential biological or behavioral responses. Women, for instance, may experience higher internal doses due to smaller airway diameters or distinct activity patterns [31]. The pronounced susceptibility observed in the 45–65-year age group aligns with the hypothesis that cumulative subclinical cardiovascular vulnerability enhances the short-term response to particulate exposures, making this demographic particularly prone to acute air pollution impacts [28]. Another explanation could be behavioral tendencies in seeking medical care, as men are generally more likely to avoid medical consultation. Women consistently utilize healthcare services more frequently than men. In Denmark, women show higher use of preventive screening and primary care services [32]. Comparable trends are evident in Saudi Arabia, as Almaqhawi et al., (2022) noted that 70.7% of women, in contrast to 61.8% of men, expressed a willingness to seek care at a healthcare center upon experiencing symptoms; furthermore, nearly half of the participants favored public healthcare facilities over private ones [33]. The observed pollutant-specific risks are biologically plausible and supported by established mechanistic pathways. BC, due to its large surface area, acts as a carrier for adsorbed metals and organic compounds, facilitating their deep penetration into the alveoli and subsequent translocation across the air–blood barrier. This process promotes oxidative stress, endothelial dysfunction, and systemic inflammation, mechanistic pathways that are well-documented in experimental studies [6,21].

Transition and industrial metals (e.g., Fe, Ni, Cr, Pb, Cu, Mn) are known to catalyze reactive oxygen species (ROS) formation through Fenton-like reactions, leading to redox imbalance and triggering vascular and autonomic dysregulation [6,21]. The short-lag responses (0–2 days) observed for BC and Ni likely reflect acute inflammatory and sympathetic activation, while the extended effects of BC up to lag 4 suggest continued oxidative injury and delayed symptom exacerbation [6,21]. Elevated inpatient risks may thus represent pollutant-triggered decompensation in individuals with pre-existing cardiopulmonary disease.

A significant proportion of our cardiovascular outcomes included cases coded for hypertension (ICD-10 I10–I15). Although hypertension is not associated with acute symptoms and therefore is an unlikely cause for seeking medical care, it is a chronic cardiovascular condition. Its inclusion is highly relevant given the established link between air pollution exposure and acute cardiovascular events. Air pollution can acutely exacerbate existing cardiovascular conditions, and elevated blood pressure can serve as a signal of this exacerbation. The sensitivity analysis, which excluded 118 hypertension-only CVD cases, demonstrated an amplification of the associations for combustion markers (BC, Pb, S, Fe). This finding suggests that these pollutants exert significant impacts even beyond those primarily driven by hypertension, indicating that air pollution can worsen outcomes in individuals with pre-existing hypertension. Given that hypertension forms a substantial proportion of CVD outcomes, its consideration is crucial for understanding the full public health burden and for informing targeted interventions for vulnerable populations [22].

The clear dominance of combustion-derived and metal-rich PM_2.5_ fractions in driving acute health responses underscores the urgency of source-specific mitigation strategies. Traffic congestion, industrial activity, and energy generation are key contributors to these pollutants in Makkah, especially during high-density periods such as the Hajj and Umrah seasons [14,23]. Implementing targeted emission controls, such as improved vehicle inspection systems, cleaner fuels, enhanced industrial filtration, and real-time air-quality alerts, could yield immediate and substantial health gains. Urban planning measures aimed at reducing congestion near Alharam, combined with targeted regulation of metal emissions, would address both the magnitude of exposure and the toxicity of the particulate matter. Importantly, our results advocate for regulatory frameworks that account not only for PM_2.5_ mass concentration but also for its chemical composition and source toxicity, aligning with recent WHO guidelines emphasizing component-specific risk assessment [34].

This study has several notable strengths. It utilized a full-year dataset with daily PM_2.5_ chemical speciation collected from five monitoring sites across Makkah, providing exceptional temporal and spatial coverage. Hospital admission data were integrated with validated diagnostic codes, ensuring clinical reliability. The application of both single- and multi-pollutant GLMs, with rigorous adjustment for potential confounders including temperature, humidity, seasonality, and day-of-week, strengthened the internal validity of findings. Stratified and sensitivity analyses further confirmed the robustness and consistency of the observed associations. Additionally, excluding elements with more than 20% of values below detection limits (e.g., Ga, Zr) minimized noise and improved estimate precision.

Despite these strengths, certain limitations should be acknowledged. The health analysis relied primarily on exposure data from a single central monitoring site (Alharam), which, although representing the highest PM_2.5_ levels and constituent concentrations, may not fully capture individual exposure variability across the city’s diverse microenvironments. This spatial simplification could lead to exposure misclassification and potential underestimation of effect sizes. Future research could enhance exposure assessment by integrating data from all monitoring sites to derive a more representative exposure distribution. Furthermore, our limited sample sizes, especially in certain stratified analyses, reduced statistical precision. Additional limitations include: (1) potential selection bias from using a single hospital (Herra Hospital), as admissions to other facilities were not captured; (2) lack of individual-level confounders (smoking, BMI, comorbidities) in our ecological design, which may affect causal inference; and (3) the 12-month study period, which is too brief to capture interannual variability or to conduct robust stratified analyses comparing Hajj and non-Hajj periods.

An important consideration is that the measured constituents, including crustal elements, trace metals, BC, and selected ions, accounted for less than half of the total PM_2.5_ mass, despite the high mean PM_2.5_ concentration of 113.6 µg/m^3^. The unmeasured fraction likely included organic carbon (OC), bound water, carbonates from desert dust, and semi-volatile species. Organic carbon, in particular, may contribute substantially to PM_2.5_ mass and has been linked to oxidative stress and particle toxicity. Therefore, the absence of these components may have limited the ability to fully characterize PM_2.5_ composition and could have led to underestimation of the true health effects associated with particulate exposure. Additionally, SO_4_^2−^ was not evaluated separately in the cardiopulmonary association models. The S variable used in the present analysis represents total elemental S measured by EDXRF, whereas sulfate is a water-soluble ion measured by ion chromatography. Future studies should examine sulfate-specific associations with cardiopulmonary outcomes in Makkah.

Finally, the ecological nature of the time-series design restricts causal inference at the individual level.

Future work should integrate expanded spatial monitoring and personal exposure studies to capture population-level heterogeneity. Applying source-apportionment and oxidative potential metrics could help disentangle the relative toxicity of dust, traffic, and industrial sources. Longer study periods would improve statistical power for rarer events and capture interannual variability, while pilgrimage-season analyses could quantify transient health burdens during extreme population density. Complementary biomarker or cohort studies are warranted to validate the mechanistic pathways of oxidative stress, systemic inflammation, and cardiovascular autonomic imbalance in local populations.

## 5. Conclusions

This study provides the first constituent-resolved evidence from Makkah linking specific PM_2.5_ components to short-term cardiopulmonary hospital admissions. Over the 12-month study period, mean PM_2.5_ concentrations were high at 113.6 µg/m^3^. Combustion-related and metal-enriched fractions, particularly BC and Pb, showed the strongest associations with adverse health outcomes. BC was associated with an 18% increase in cardiovascular ER/OP visits (aRR = 1.18; 95% CI: 1.08–1.29) and a 25% increase in inpatient admissions (aRR = 1.25; 95% CI: 1.07–1.46), while Pb remained independently associated with admissions in multi-pollutant models (aRR = 1.15; 95% CI: 1.01–1.32). In contrast, crustal elements showed no significant associations. The strongest effects occurred within 0–2 days of exposure, supporting rapid inflammatory and oxidative stress pathways. Risks were more pronounced among females and adults aged 45–65 years, indicating important susceptibility patterns. These findings suggest that air-quality management in Makkah should move beyond total PM_2.5_ mass and prioritize targeted control of traffic and industrial combustion sources, constituent-based air-quality standards, and public health interventions for vulnerable groups during peak pollution periods, including the Hajj season.

## Figures and Tables

**Table 1:** Characteristics of PM_2.5_ constituents (µg/m^3^) measured across monitoring sites in Makkah.

Component	Alharam (Primary Site)	Al-Hajj	Alshoqiyah	Herra Hospital	Ar Rusayfah
PM_2.5_	113 (458; 83.8)	59.6 (254; 31.3)	59.3 (361; 26.8)	63.7 (342; 29.3)	75.9 (285; 39.0)
Crustal Material					
Al	4.47 (15.6; 3.84)	2.85 (9.28; 2.32)	2.63 (14.3; 1.89)	1.78 (7.76; 1.22)	3.47 (10.3; 2.05)
Ca	11.4 (43.1; 12.5)	3.81 (12.3; 2.91)	4.12 (13.7; 3.20)	3.09 (10.9; 2.41)	5.02 (15.4; 3.82)
K	1.31 (5.93; 1.18)	0.220 (0.640; 0.090)	0.130 (0.370; 0.100)	0.270 (1.20; 0.120)	0.230 (0.440; 0.110)
Fe	5.99 (32.0; 6.18)	2.71 (17.2; 2.07)	3.58 (31.3; 2.53)	2.01 (9.06; 1.18)	4.90 (21.3; 3.38)
Mg	1.19 (4.92; 1.17)	0.180 (0.430; 0.130)	0.170 (0.540; 0.130)	0.190 (1.11; 0.160)	0.270 (0.830; 0.130)
Ti	0.520 (2.51; 0.520)	0.050 (0.120; 0.040)	0.060 (0.190; 0.050)	0.050 (0.150; 0.040)	0.070 (0.220; 0.060)
Si	12.1 (45.7; 13.0)	5.54 (24.4; 4.62)	48.6 (271; 35.5)	4.52 (21.7; 2.79)	10.1 (32.4; 5.78)
Sea Spray					
Na	0.960 (2.09; 0.520)	0.690 (2.05; 0.590)	0.710 (1.83; 0.660)	0.970 (10.6; 0.920)	0.890 (1.83; 0.460)
Cl	0.850 (2.75; 1.20)	0.230 (1.28; 0.210)	0.210 (0.970; 0.260)	0.630 (15.9; 0.270)	0.550 (4.50; 0.490)
Fossil-Fuel Combustion					
Ni	0.024 (0.093; 0.020)	0.011 (0.051; 0.005)	0.013 (0.072; 0.006)	0.009 (0.022; 0.005)	0.016 (0.063; 0.011)
S	4.44 (11.7; 2.73)	8.91 (27.1; 7.07)	9.35 (34.1; 7.23)	10.1 (24.3; 7.08)	2.91 (7.50; 1.77)
NO_3_^−^	4.00 (15.0; 2.66)	1.54 (3.49; 1.00)	1.71 (9.86; 1.16)	1.88 (5.82; 1.89)	4.11 (13.7; 3.06)
NH_4_^+^	1.50 (7.00; 2.31)	1.74 (6.60; 1.82)	2.08 (7.47; 2.79)	1.73 (6.70; 1.19)	1.31 (4.09; 1.23)
BC	2.85 (8.10; 1.80)	1.70 (3.50; 1.05)	1.28 (5.00; 1.00)	1.95 (5.00; 1.25)	1.46 (3.20; 0.900)
Industrial Emissions					
Cr	0.022 (0.095; 0.020)	0.011 (0.056; 0.006)	0.012 (0.090; 0.008)	0.008 (0.028; 0.005)	0.015 (0.062; 0.012)
Mn	0.130 (0.680; 0.120)	0.056 (0.303; 0.045)	0.076 (0.630; 0.059)	0.043 (0.161; 0.030)	0.104 (0.450; 0.073)
Cu	0.035 (0.110; 0.027)	0.026 (0.084; 0.011)	0.027 (0.127; 0.013)	0.012 (0.046; 0.008)	0.020 (0.093; 0.015)
Zn	0.130 (0.530; 0.100)	0.043 (0.157; 0.019)	0.042 (0.171; 0.021)	0.029 (0.082; 0.016)	0.047 (0.243; 0.025)
Pb	0.065 (1.20; 0.050)	0.058 (0.952; 0.043)	0.092 (2.84; 0.018)	0.030 (0.285; 0.022)	0.081 (0.905; 0.067)

Values are presented as Mean (Maximum; IQR); PM_2.5_—particulate matter ≤ 2.5 µm; BC—black carbon; nitrate—NO_3_^−^; NH_4_^+^—ammonium.

**Table 2:** Demographic and clinical characteristics of hospital admissions (*n* = 584).

Characteristic	Cardiovascular (*n* = 300)	Pulmonary (*n* = 284)	Total (*n* = 584)
Sex			
Female, *n* (%)	170 (56.7%)	130 (45.8%)	300 (51.4%)
Male, *n* (%)	130 (43.3%)	154 (54.2%)	284 (48.6%)
Age group			
<45 years, *n* (%)	42 (14.0%)	159 (56.0%)	201 (34.4%)
45–65 years, *n* (%)	150 (50.0%)	100 (35.2%)	250 (42.8%)
>65 years, *n* (%)	108 (36.0%)	25 (8.8%)	133 (22.8%)
Visit type			
ER/OP visits, *n* (%)	273 (91.0%)	216 (76.1%)	489 (83.7%)
Inpatient visits, *n* (%)	27 (9.0%)	68 (23.9%)	95 (16.3%)
Disease Type, *n* (%)			
COPD		105	17.98%
Bronchopneumonia		179	30.65%
Unstable angina	66		11.3%
Hypertension	233		39.89%
Other	1		0.17%

“ER/OP” includes emergency and outpatient admissions; Hypertension is classified under cardiovascular disease codes based on the ICD-10; For sensitivity analyses, hypertension-only cases (*n* = 118) were excluded and models rerun.

**Table 3:** Adjusted relative risks (aRRs; 95% CI) for CVD-related ER visits and hospital admissions associated with PM_2.5_ constituents in Makkah.

PM_2.5_ Constituent	Disease Type	Outcome	Single Model aRR (95% CI)	Multiple Model aRR (95% CI)
Al	CVD	ER-OP	1.03 (0.95–1.12)	1.00 (0.91–1.10)
		Inpatient	1.08 (0.93–1.26)	1.04 (0.89–1.22)
	Pulmonary	ER-OP	1.06 (0.97–1.17)	1.02 (0.93–1.12)
		Inpatient	1.11 (0.96–1.29)	1.07 (0.91–1.26)
BC	CVD	ER-OP	1.18 (1.08–1.29)	1.12 (1.01–1.25)
		Inpatient	1.25 (1.07–1.46)	1.19 (1.02–1.38)
	Pulmonary	ER-OP	1.14 (1.04–1.25)	1.09 (0.99–1.20)
		Inpatient	1.20 (1.03–1.40)	1.15 (1.00–1.33)
Ni	CVD	ER-OP	1.24 (0.89–1.72)	1.019 (1.008–1.030)
		Inpatient	1.017 (1.007–1.027)	1.028 (1.017–1.039)
	Pulmonary	ER-OP	1.15 (1.09–1.76)	1.37 (1.09–1.48)
		Inpatient	1.36 (1.14–1.59)	1.02 (0.91–1.15)
NO_3_^−^	CVD	ER-OP	1.06 (0.98–1.14)	1.04 (0.95–1.13)
		Inpatient	1.12 (0.97–1.29)	1.07 (0.91–1.24)
	Pulmonary	ER-OP	1.07 (0.99–1.16)	1.03 (0.94–1.13)
		Inpatient	1.10 (0.95–1.27)	1.05 (0.90–1.22)
S	CVD	ER-OP	1.15 (1.06–1.24)	1.10 (1.00–1.21)
		Inpatient	1.22 (1.06–1.40)	1.17 (1.01–1.36)
	Pulmonary	ER-OP	1.13 (1.03–1.24)	1.09 (0.99–1.20)
		Inpatient	1.20 (1.03–1.39)	1.15 (0.99–1.33)
Fe	CVD	ER-OP	1.11 (1.01–1.23)	1.07 (0.96–1.19)
		Inpatient	1.18 (1.02–1.37)	1.12 (0.96–1.32)
	Pulmonary	ER-OP	1.10 (1.00–1.21)	1.06 (0.95–1.18)
		Inpatient	1.15 (0.99–1.34)	1.10 (0.94–1.29)
Mg	CVD	ER-OP	1.14 (1.04–1.25)	1.10 (1.00–1.21)
		Inpatient	1.21 (1.05–1.39)	1.16 (1.01–1.34)
	Pulmonary	ER-OP	1.12 (1.02–1.23)	1.07 (0.97–1.19)
		Inpatient	1.18 (1.01–1.37)	1.13 (0.98–1.31)
Ti	CVD	ER-OP	1.20 (1.11–1.30)	0.83 (0.65–1.09)
		Inpatient	1.28 (1.12–1.46)	0.92 (0.81–1.09)
	Pulmonary	ER-OP	1.16 (1.06–1.26)	1.03 (0.91–1.14)
		Inpatient	1.24 (1.09–1.41)	1.08 (0.89–1.10)
Pb	CVD	ER-OP	1.16 (1.05–1.28)	1.11 (0.99–1.23)
		Inpatient	1.21 (1.06–1.38)	1.15 (1.01–1.32)
	Pulmonary	ER-OP	1.13 (1.02–1.25)	1.09 (0.98–1.21)
		Inpatient	1.18 (1.03–1.35)	1.12 (0.98–1.29)
NH_4_^+^	CVD	ER-OP	1.10 (1.02–1.18)	1.07 (0.99–1.15)
		Inpatient	1.12 (0.98–1.28)	1.09 (0.95–1.25)
	Pulmonary	ER-OP	0.87 (0.63–1.13)	0.98 (0.74–1.21)
		Inpatient	1.16 (1.05–1.32)	1.00 (0.83–1.12)

**Table 4:** Sensitivity analysis hypertension cases excluded (aRRs, 95% CI) per IQR increase in PM_2.5_ constituents.

PM_2.5_ Constituent	Outcome	Single Model aRR (95% CI)	Multiple Model aRR (95% CI)
Al	ER-OP	1.12 (0.78–1.63)	1.02 (0.67–1.55)
	Inpatient	1.38 (0.71–2.76)	1.21 (0.60–2.48)
BC	ER-OP	2.12 (1.44–3.17)	1.68 (1.07–2.74)
	Inpatient	2.70 (1.34–5.42)	2.20 (1.10–4.26)
Ni	ER-OP	2.65 (0.61–11.40)	1.10 (1.05–1.15)
	Inpatient	1.09 (1.04–1.14)	1.14 (1.09–1.20)
NO_3_^−^	ER-OP	1.32 (0.92–1.83)	1.20 (0.80–1.75)
	Inpatient	1.68 (0.88–3.15)	1.36 (0.67–2.65)
S	ER-OP	1.89 (1.31–2.64)	1.55 (1.01–2.36)
	Inpatient	2.46 (1.32–4.54)	2.04 (1.07–3.98)
Fe	ER-OP	1.62 (1.06–2.55)	1.36 (0.84–2.20)
	Inpatient	2.12 (1.10–4.11)	1.67 (0.84–3.49)
Mg	ER-OP	1.82 (1.20–2.74)	1.55 (1.01–2.36)
	Inpatient	2.38 (1.26–4.40)	1.95 (1.06–3.73)
Ti	ER-OP	2.28 (1.61–3.26)	0.45 (0.16–1.50)
	Inpatient	3.04 (1.67–5.46)	0.71 (0.40–1.49)
Pb	ER-OP	1.95 (1.25–3.04)	1.62 (0.97–2.55)
	Inpatient	2.36 (1.31–4.25)	1.88 (1.06–3.48)
NH_4_^+^	ER-OP	1.55 (1.10–2.12)	1.36 (0.97–1.89)
	Inpatient	1.68 (0.92–3.05)	1.48 (0.80–2.73)

Models: single-pollutant negative-binomial GLMs adjusted for temperature, humidity, seasonality, day of week, and public holidays.

**Table 5:** Multi-Pollutant Adjusted Relative Risks (aRRs; 95% CI) for Cardiopulmonary Hospital Admissions Associated with PM_2.5_ Constituents.

PM_2.5_ Constituent	Outcome	Visit Type	Model Specification (Co-Pollutants Included)	aRR (95% CI)	*p*-Value
BC	CVD	ER/OP	+Pb + S + Ni + Fe	1.12 (1.01–1.25)	0.03
	CVD	Inpatient	+Pb + S + Ni + Fe	1.19 (1.02–1.38)	0.03
	Pulmonary	ER/OP	+Pb + S + Ni + Fe	1.09 (0.99–1.20)	0.08
	Pulmonary	Inpatient	+Pb + S + Ni + Fe	1.15 (1.00–1.33)	0.05
Pb	CVD	ER/OP	+BC + S + Ni + Fe	1.11 (0.99–1.23)	0.07
	CVD	Inpatient	+BC + S + Ni + Fe	1.15 (1.01–1.32)	0.04
	Pulmonary	ER/OP	+BC + S + Ni + Fe	1.09 (0.98–1.21)	0.11
	Pulmonary	Inpatient	+BC + S + Ni + Fe	1.12 (0.98–1.29)	0.09
Ni	CVD	ER/OP	+BC + S + Pb	1.019 (1.008–1.030)	<0.01
	CVD	Inpatient	+BC + S + Pb	1.028 (1.017–1.039)	<0.01
	Pulmonary	ER/OP	+BC + S + Pb	1.37 (1.09–1.48)	<0.01
	Pulmonary	Inpatient	+BC + S + Pb	1.02 (0.91–1.15)	0.73
S	CVD	ER/OP	+BC + Pb + Ni + Fe	1.10 (1.00–1.21)	0.05
	CVD	Inpatient	+BC + Pb + Ni + Fe	1.17 (1.01–1.36)	0.04
	Pulmonary	ER/OP	+BC + Pb + Ni + Fe	1.09 (0.99–1.20)	0.08
	Pulmonary	Inpatient	+BC + Pb + Ni + Fe	1.15 (0.99–1.33)	0.06
Fe	CVD	ER/OP	+BC + Pb + S + Mg	1.07 (0.96–1.19)	0.22
	CVD	Inpatient	+BC + Pb + S + Mg	1.12 (0.96–1.32)	0.16
	Pulmonary	ER/OP	+BC + Pb + S + Mg	1.06 (0.95–1.18)	0.29
	Pulmonary	Inpatient	+BC + Pb + S + Mg	1.10 (0.94–1.29)	0.23
Mg	CVD	ER/OP	+BC + Fe + Ti	1.10 (1.00–1.21)	0.05
	CVD	Inpatient	+BC + Fe + Ti	1.16 (1.01–1.34)	0.04
	Pulmonary	ER/OP	+BC + Fe + Ti	1.07 (0.97–1.19)	0.18
	Pulmonary	Inpatient	+BC + Fe + Ti	1.13 (0.98–1.31)	0.09
Ti	CVD	ER/OP	+BC + Fe + Mg	0.83 (0.65–1.09)	0.18
	CVD	Inpatient	+BC + Fe + Mg	0.92 (0.81–1.09)	0.34
	Pulmonary	ER/OP	+BC + Fe + Mg	1.03 (0.91–1.14)	0.62
	Pulmonary	Inpatient	+BC + Fe + Mg	1.08 (0.89–1.10)	0.44
Al	CVD	ER/OP	+Si + Ca + Fe	1.00 (0.91–1.10)	0.98
	CVD	Inpatient	+Si + Ca + Fe	1.04 (0.89–1.22)	0.62
	Pulmonary	ER/OP	+Si + Ca + Fe	1.02 (0.93–1.12)	0.69
	Pulmonary	Inpatient	+Si + Ca + Fe	1.07 (0.91–1.26)	0.42
NO_3_^−^	CVD	ER/OP	+NH_4_^+^ + S	1.04 (0.95–1.13)	0.42
	CVD	Inpatient	+NH_4_^+^ + S	1.07 (0.91–1.24)	0.41
	Pulmonary	ER/OP	+NH_4_^+^ + S	1.03 (0.94–1.13)	0.51
	Pulmonary	Inpatient	+NH_4_^+^ + S	1.05 (0.90–1.22)	0.52
NH_4_^+^	CVD	ER/OP	+NO_3_^−^ + S	1.07 (0.99–1.15)	0.09
	CVD	Inpatient	+NO_3_^−^ + S	1.09 (0.95–1.25)	0.22
	Pulmonary	ER/OP	+NO_3_^−^ + S	0.98 (0.74–1.21)	0.86
	Pulmonary	Inpatient	+NO_3_^−^ + S	1.00 (0.83–1.12)	0.98

**Table 6:** Comparison of PM_2.5_ constituent effects on cardiopulmonary admissions across similar environments.

Study (Year)	Location	Mean PM_2.5_ (µg/m^3^)	Key Constituents Assessed	Main Findings	Effect Size (aRR per IQR)
Present study (2026)	Makkah, Saudi Arabia	113.6	BC, Pb, Ni, S, Fe, Crustals	BC and Pb strongest predictors; crustal elements null	BC: 1.18–1.25; Pb: 1.16–1.21
Nayebare et al. (2017) [3]	Jeddah, Saudi Arabia	68.5	BC, SO_4_^2−^, NO_3_^−^, NH_4_^+^	BC and secondary aerosols associated with cardiopulmonary admissions	BC: 1.12–1.18
Milibari et al. (2025) [23]	Makkah, Saudi Arabia	95.2	PM_2.5_ mass only	PM_2.5_ mass associated with CVD mortality during Hajj	PM_2.5_: 1.08–1.15
Wu et al. (2025) [7]	Urumqi, China	89.4	BC, Pb, Ni, Cr, As	BC and metals associated with cardiometabolic admissions	BC: 1.14–1.22; Pb: 1.09–1.16
You et al. (2023) [27]	Ganzhou, China	48.6	Pb, Ni, Cr, As	Pb and Ni increased circulatory admissions	Pb: 1.12–1.19; Ni: 1.10–1.15
Feyisetan et al. (2025) [25]	Abuja, Nigeria	78.3	BC, S, heavy metals	Traffic and industrial components, not dust, drove health risk	BC: 1.15–1.20
Grivas et al. (2019) [29]	Athens, Greece	28.4	BC, S, Ni	BC and S from traffic and fuel combustion linked to morbidity	BC: 1.06–1.12

## Data Availability

The authors confirm that the data supporting the findings of this study are available within the article and the posted Supplementary data.

## Supplementary Materials

The following supporting information can be downloaded at: https://www.mdpi.com/article/10.3390/toxics14050449/s1, Figure S1: Adjusted RRs of PM_2.5_ constituents by disease types (CVD vs. Pulmonary) and visit types (ER-OP vs. Inpatient in males and females; Figure S2: Adjusted RRs of PM_2.5_ constituents by disease types (CVD vs. Pulmonary) and visit types (ER-OP vs. Inpatient by age group); Figure S3: Distribution of CVD and pulmonary visits by age group and hospital admission types; Figure S4: Distribution of CVD subtype by age group and hospital admission types; Figure S5: Distribution of pulmonary subtype by age group and hospital admission types; Figure S6: Seasonal variation of PM_2.5_ components during Hajj season and other seasons; Figure S7: (A) single pollutant model and Adjusted RRs of PM_2.5_ Constituents-Pulmonary (B) Single pollutant model Adjusted RRs of PM_2.5_ Constituents-CVD including HTN; Figure S8: independent effects of BC and Pb in single and multi-pollutant model for CVD including HTN outcome (Inpatient); Figure S9: Seasonal forest plot for BC and CVD inpatient admissions; Table S1: Pearson Correlation Coefficients (r) among PM_2.5_ Constituents across Seasons at Al-Haram, Makkah; Figure S10: line graph for the effects of BC and Ni Inpatients and ER/OP at different lag days (lag0–lag4).
