# Supplementary material for: From Mass to Molecules: PM2.5 Constituents and Cardiopulmonary Admissions in Makkah
Source: Toxics. 2026 May 21;14(5):449. doi: 10.3390/toxics14050449 (PMC13211484; doi:10.3390/toxics14050449)
Supplement: Supplementary file 1 [file toxics-14-00449-s001.zip › toxics-4245873-supplementary.pdf]

Table S1. Pearson Correlation Coefficients (r) among $PM_{2.5}$ Constituents across Seasons at Al-Haram, Makkah.

$PM_{2.5}$ Components Pair	Whole Year (r)	Spring (r)	Summer (r)	Autumn (r)	Winter (r)
**Al–Si**	0.962**	0.999**	0.958**	0.833**	0.972**
**Al–Fe**	0.929**	0.938**	0.938**	0.839**	0.969**
**Al–Ti**	0.932**	0.948**	0.949**	0.833**	0.969**
**Al–Ca**	0.876**	0.968**	0.850**	0.798**	0.955**
**Fe–Ti**	0.996**	0.998**	0.994**	0.996**	0.998**
**Mg–Ca**	0.932**	0.971**	0.930**	0.983**	0.970**
**Ni–Cr**	0.906**	0.936**	0.916**	0.937**	0.943**
**Ni–Pb**	0.913**	0.921**	0.915**	0.967**	0.943**
**Ni–Zn**	0.886**	0.906**	0.896**	0.780**	0.874**
**Cr–Pb**	0.925**	0.953**	0.953**	0.937**	0.866**
**Cr–Ni**	0.955**	0.936**	0.916**	0.937**	0.943**
**BC–S**	0.407**	0.429**	0.418**	0.247*	0.464**
**BC–$PM_{2.5}$**	0.643**	0.768**	0.725**	0.697**	0.733**
**S–NO_3^-**	0.332**	0.315*	0.661**	0.232	0.585**
**NO_3^-–NH_4^+**	0.666**	0.775**	0.673**	0.807**	0.585**
**S–NH_4^+**	–0.423**	0.540**	0.721*	0.382*	0.299
**$PM_{2.5}$–Cr**	0.927**	0.969**	0.949**	0.917**	0.505**
**$PM_{2.5}$–Ni**	0.906**	0.936**	0.916**	0.928**	0.501**
**$PM_{2.5}$–Mn**	0.938**	0.961**	0.962**	0.947**	0.552**
**$PM_{2.5}$–Pb**	0.904**	0.922**	0.962**	0.935**	0.508**
**$PM_{2.5}$–BC**	0.643**	0.768**	0.725**	0.697**	0.733**
**Pb–Zn**	0.322**	0.066	0.517**	0.052	0.345*
**Pb–NH_4^+**	–0.438**	–0.456**	0.961**	–0.236	–0.379
**Zn–Cu**	0.797**	0.909**	0.909**	0.651**	0.872**
**Mn–Si**	0.955**	0.935**	0.955**	0.980**	0.960**
**Si–Ti**	0.960**	0.952**	0.955**	0.991**	0.991**
**NH_4^+–Metals (mean)**	–0.38	–0.58	–0.46	–0.24	–0.32

Pearson's r coefficients calculated from daily concentrations of $PM_{2.5}$ components (n = 365 for annual; n ≈ 90 per season).
*Significance: $p < 0.01$, $p < 0.05$.
Bold values (**) indicate strong correlation (r ≥ 0.7).
Negative values represent anti-association (typically for NH_4^+ vs. Trace metals).
Data show consistently high inter-correlations among crustal elements, moderate correlations among industrial metals, and distinct secondary aerosol signatures in summer and winter.*

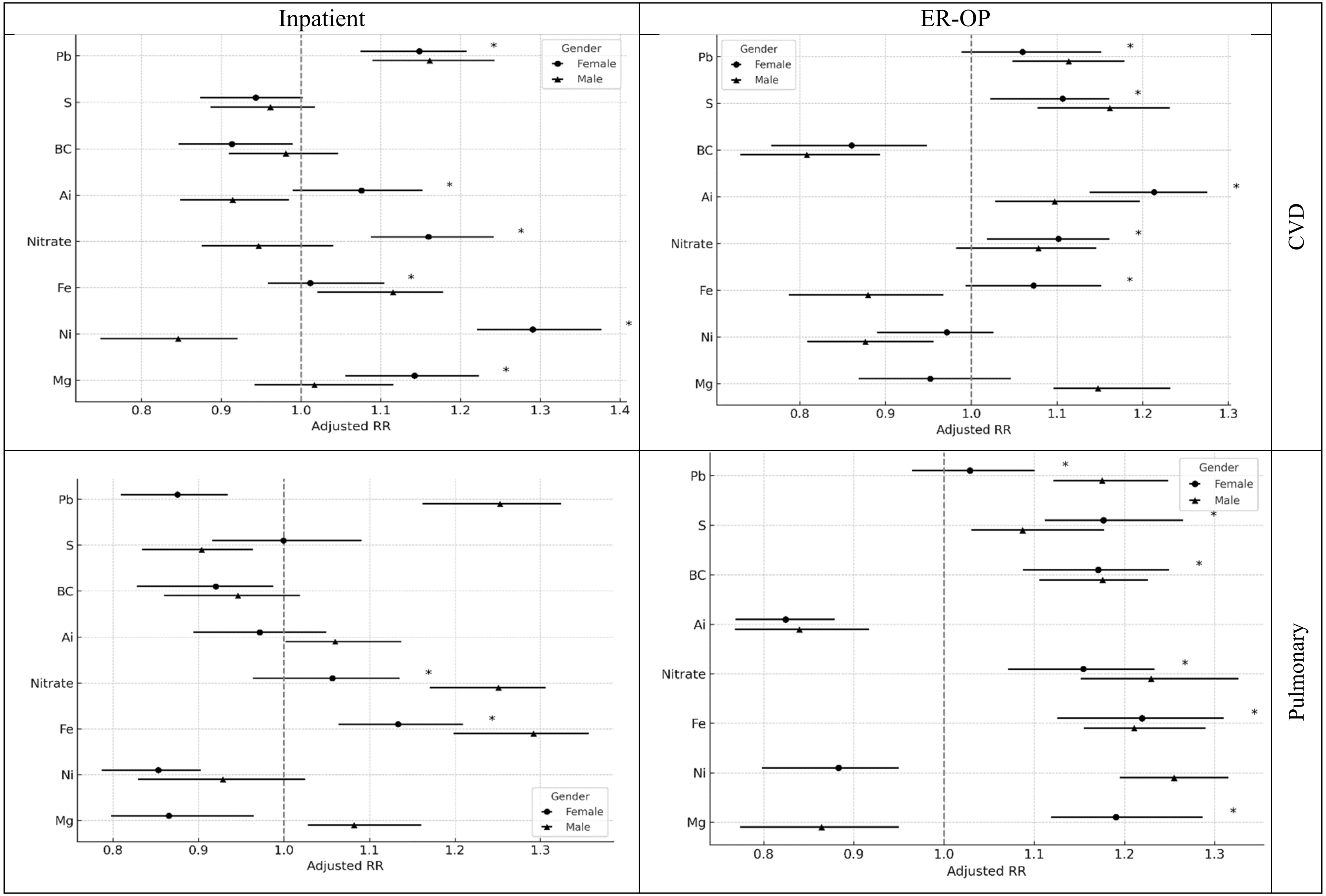

Inpatient
ER-OP
CVD
Pulmonary
Gender
Female
Male
Pb
S
BC
Ai
Nitrate
Fe
Ni
Mg
Adjusted RR
0.8
0.9
1.0
1.1
1.2
1.3
1.4
*

Fig. S1. Adjusted RRs of $PM_{2.5}$ constituents by disease types (CVD vs. Pulmonary) and visit types (ER-OP vs. Inpatient in males and females

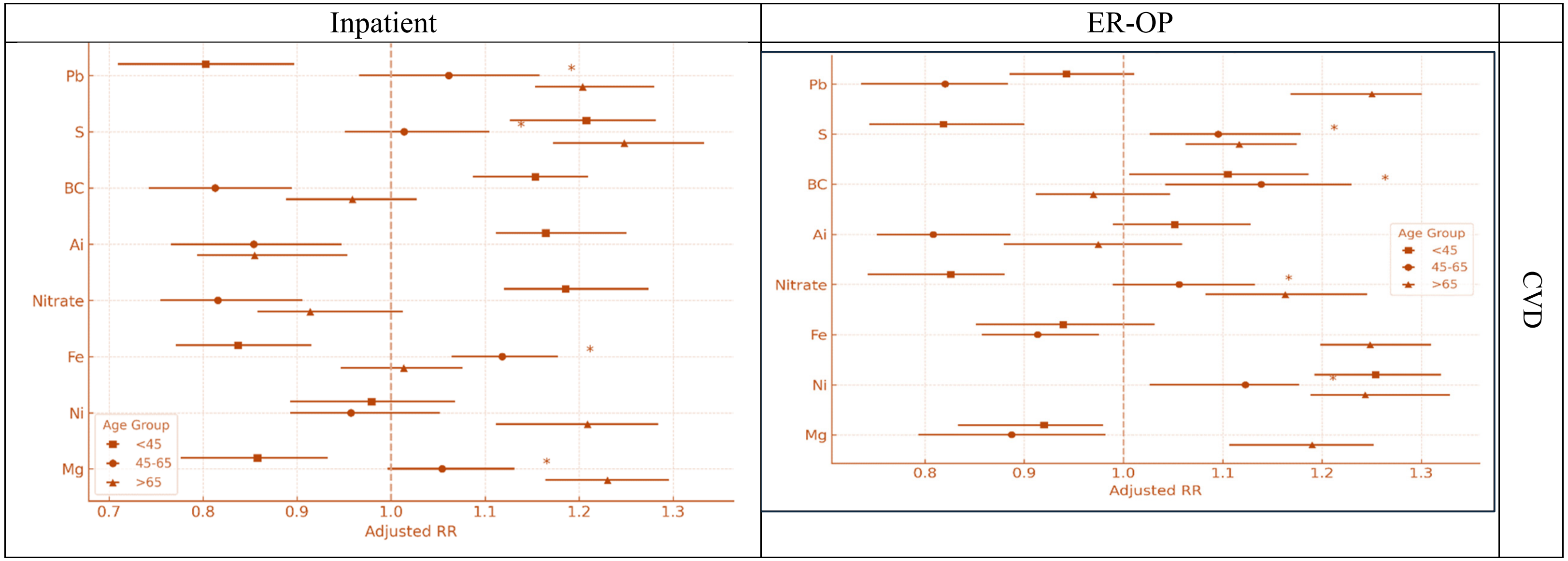

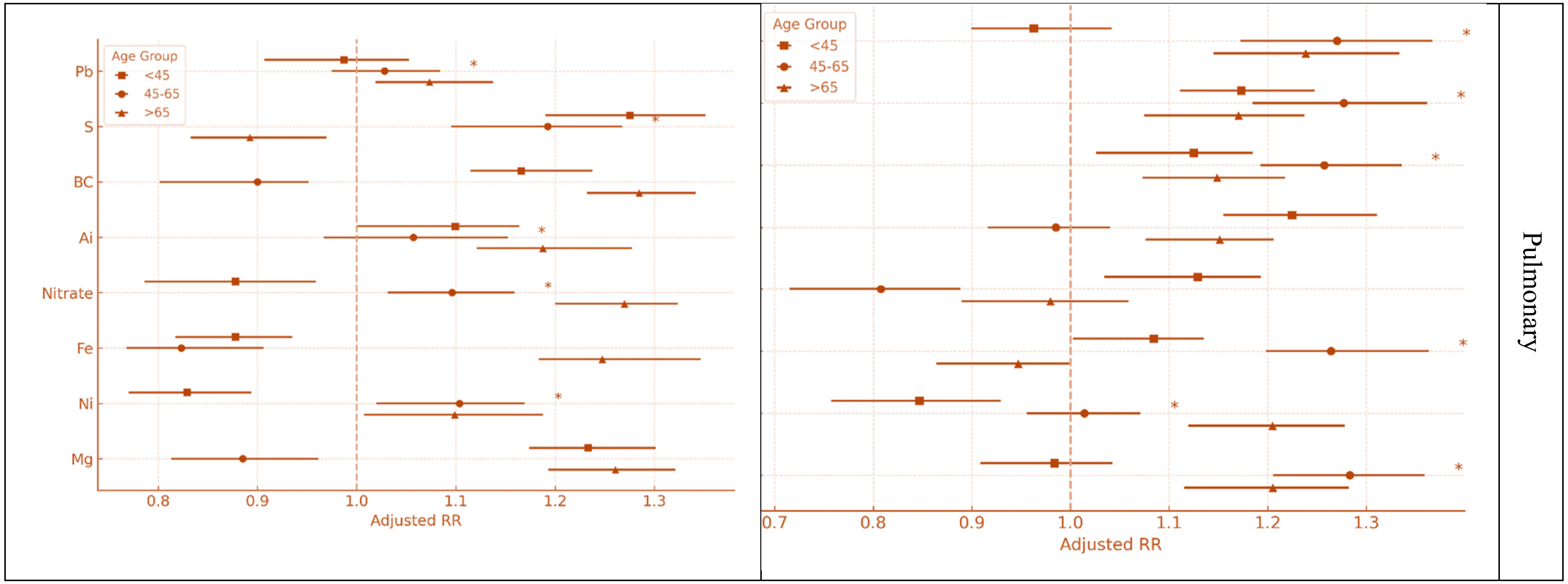

Fig. S2. Adjusted RRs of $PM_{2.5}$ constituents by disease types (CVD vs. Pulmonary) and visit types (ER-OP vs. Inpatient by age group).

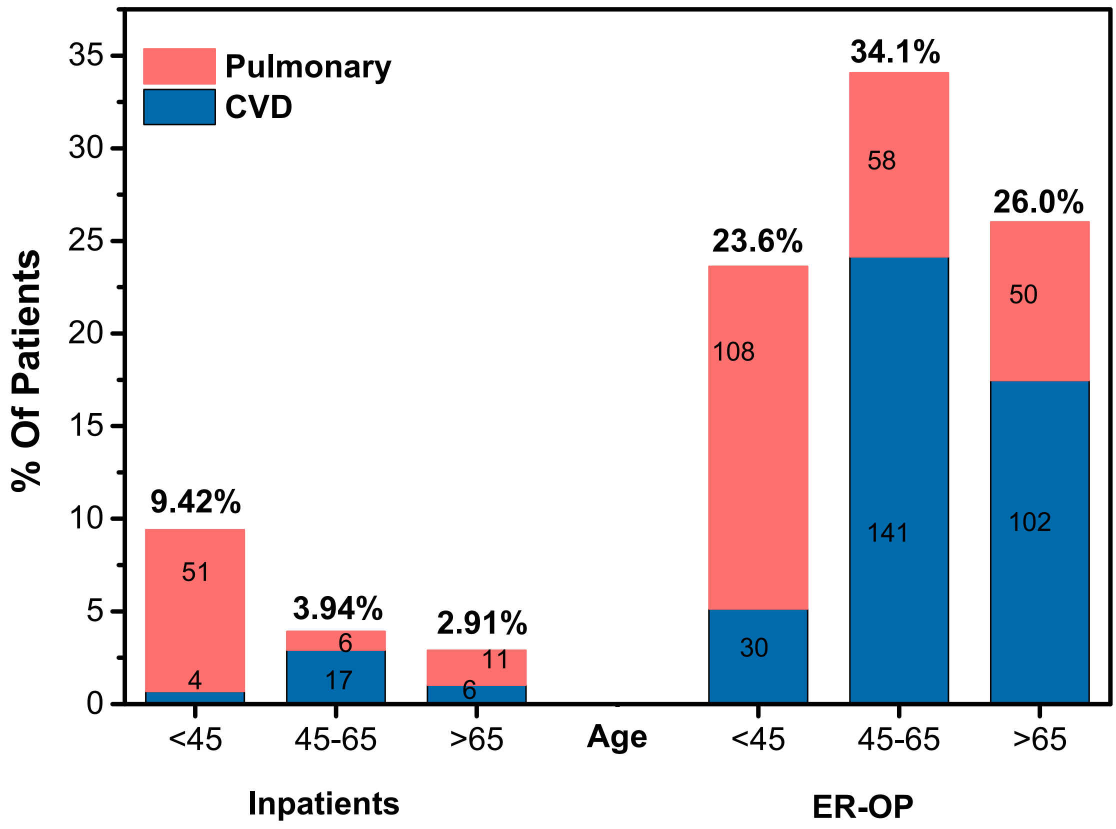

Fig S3. Distribution of CVD and pulmonary visits by age group and hospital admission types

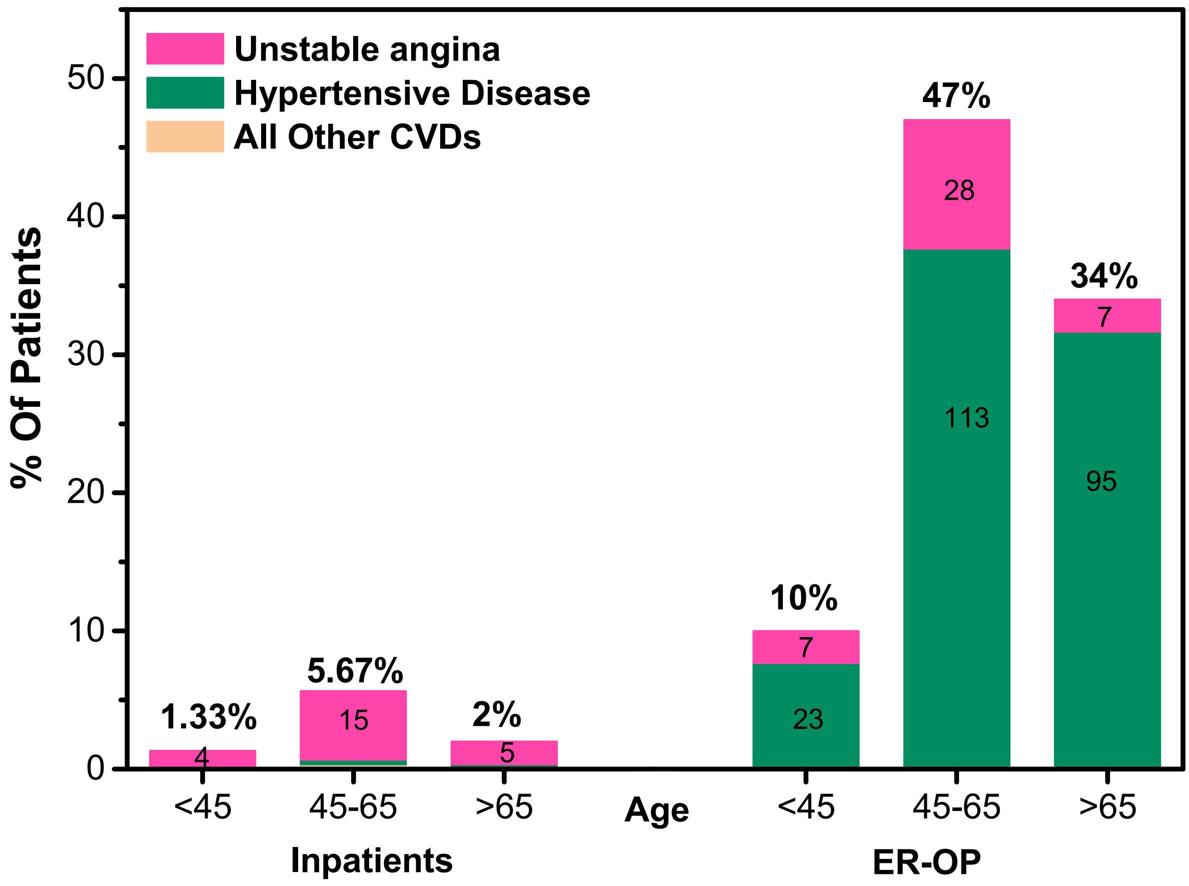

Fig. S4. Distribution of CVD subtype by age group and hospital admission types

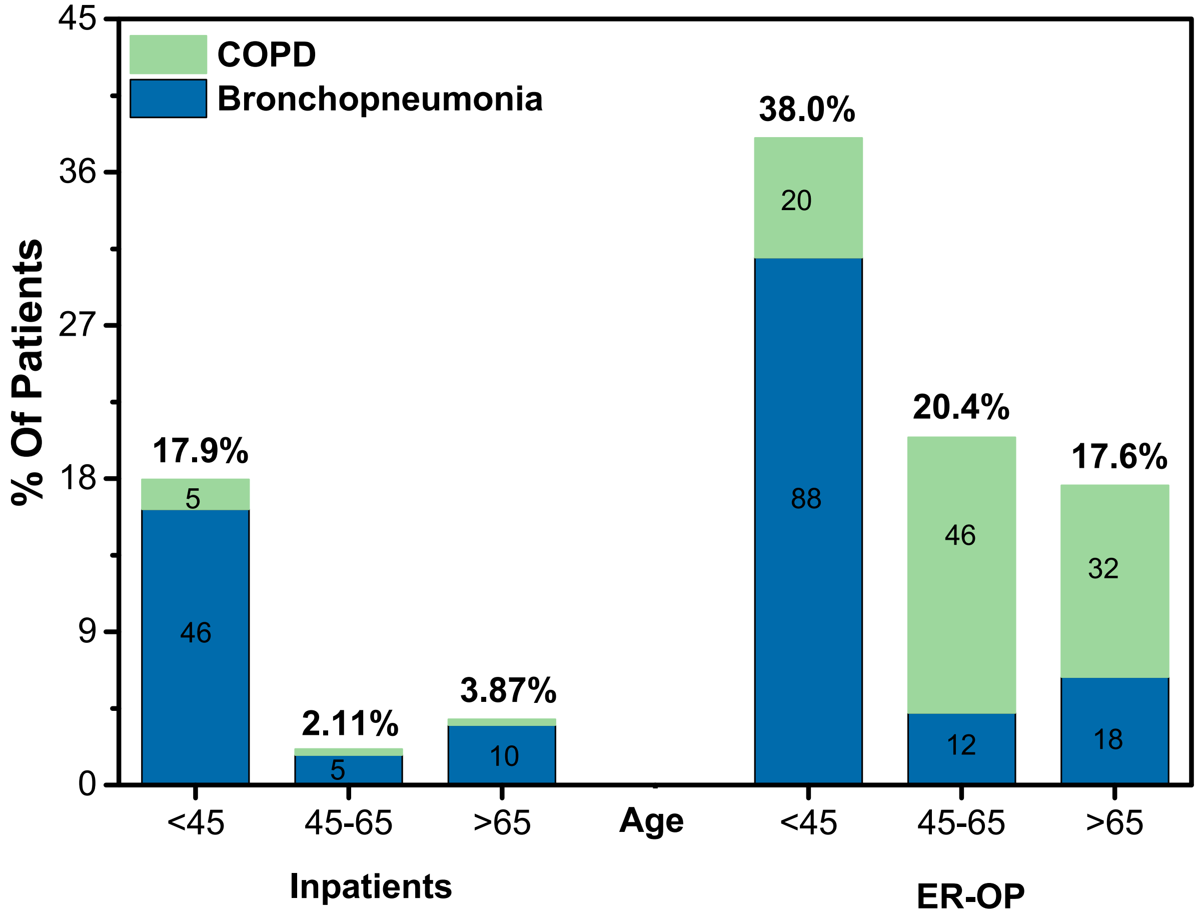

Fig.S5 Distribution of pulmonary subtype by age group and hospital admission types

Fig S6 Seasonal variation of $PM_{2.5}$ components during Hajj season and other seasons

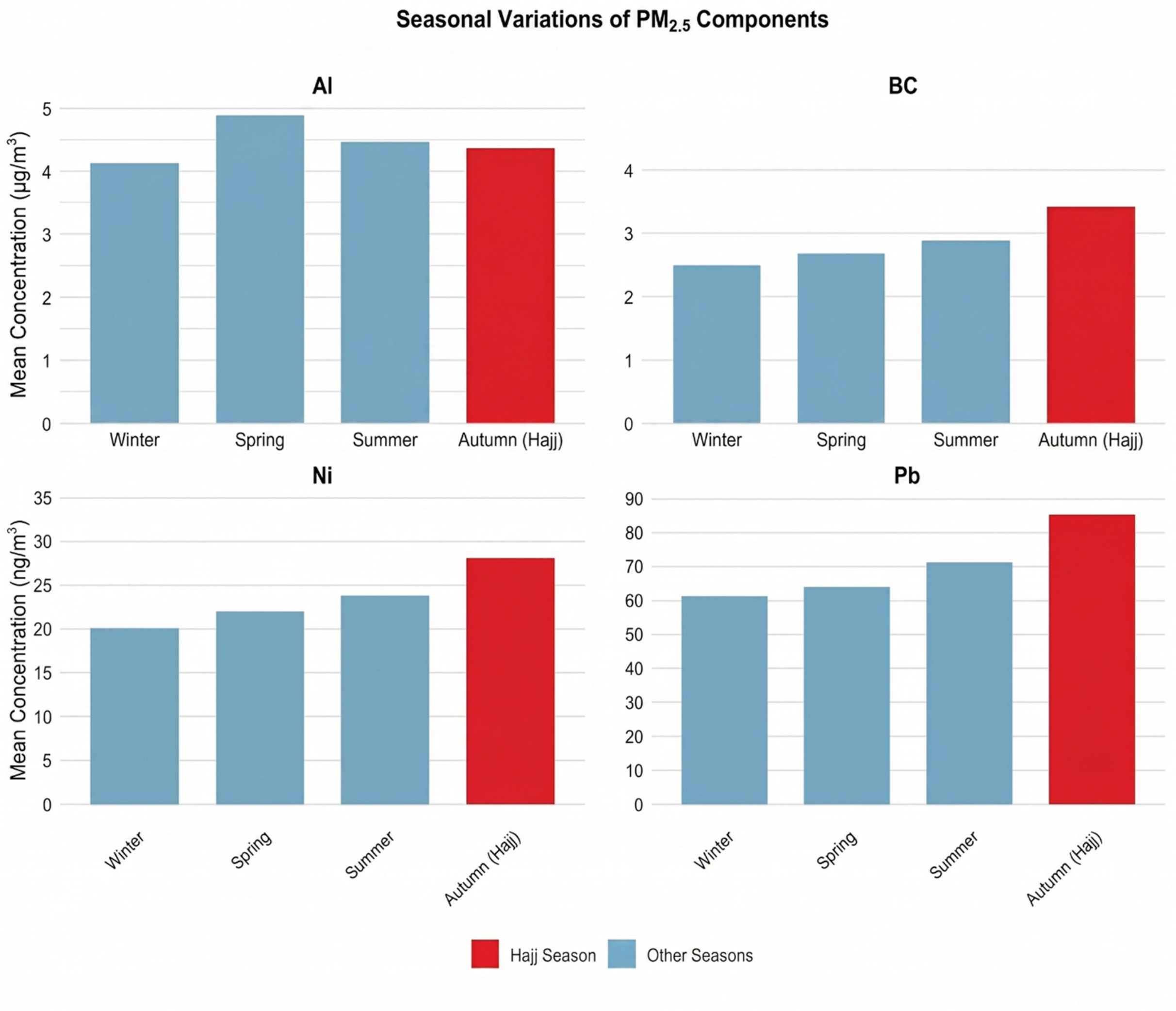

(A)

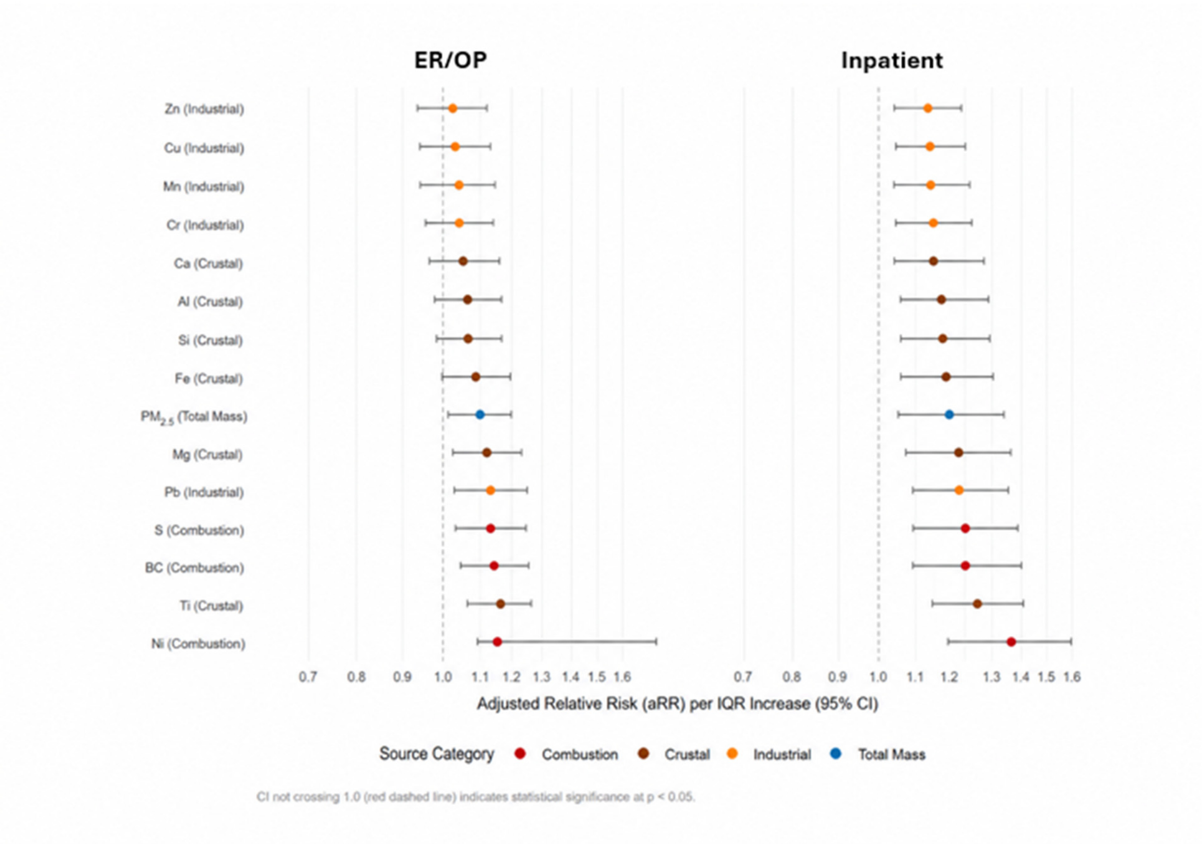

ER/OP
Inpatient
Zn (Industrial)
Cu (Industrial)
Mn (Industrial)
Cr (Industrial)
Ca (Crustal)
Al (Crustal)
Si (Crustal)
Fe (Crustal)
PM2.5 (Total Mass)
Mg (Crustal)
Pb (Industrial)
S (Combustion)
BC (Combustion)
Ti (Crustal)
Ni (Combustion)
0.7 0.8 0.9 1.0 1.1 1.2 1.3 1.4 1.5 1.6
0.7 0.8 0.9 1.0 1.1 1.2 1.3 1.4 1.5 1.6
Adjusted Relative Risk (aRR) per IQR Increase (95% CI)
Source Category
Combustion
Crustal
Industrial
Total Mass
CI not crossing 1.0 (red dashed line) indicates statistical significance at p < 0.05.

(B)

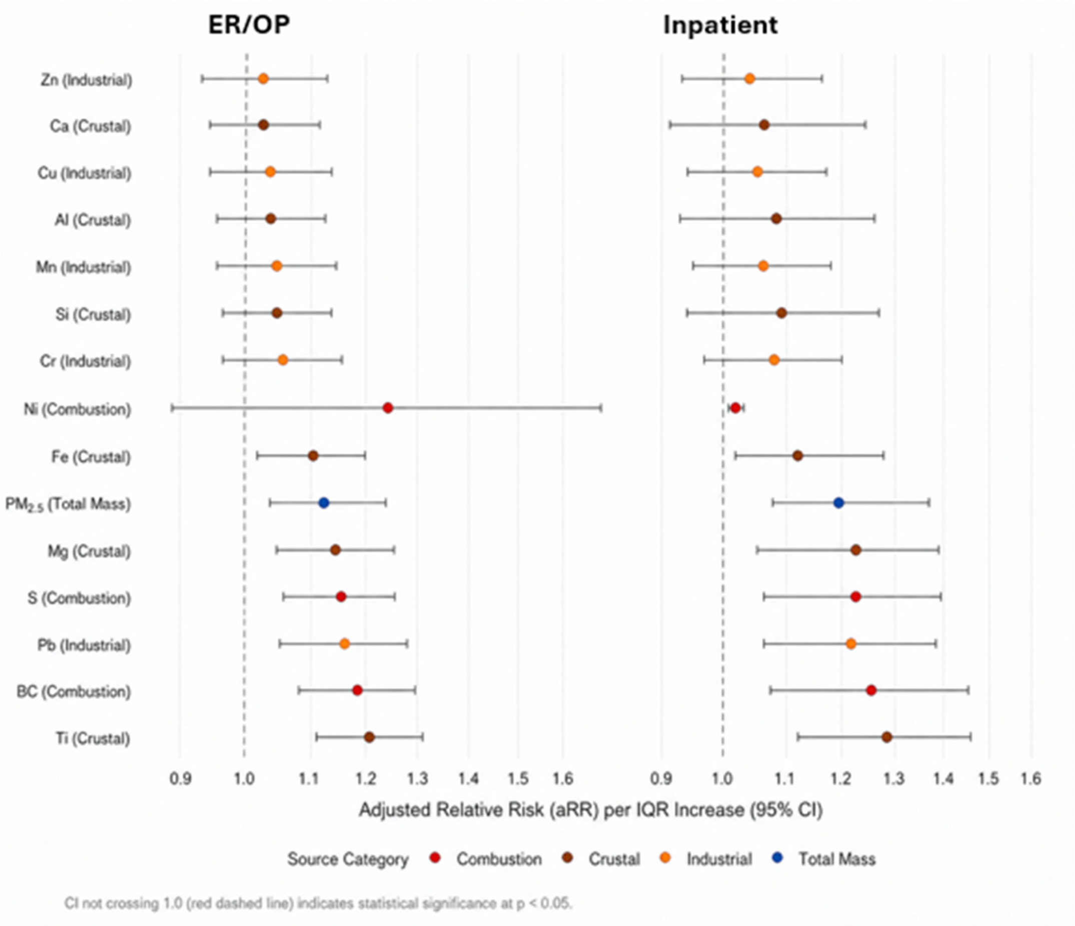

Figure S7 (A) single pollutant model and Adjusted RRs of $PM_{2.5}$ Constituents-Pulmonary (B) Single pollutant model Adjusted RRs of $PM_{2.5}$ Constituents-CVD including HTN

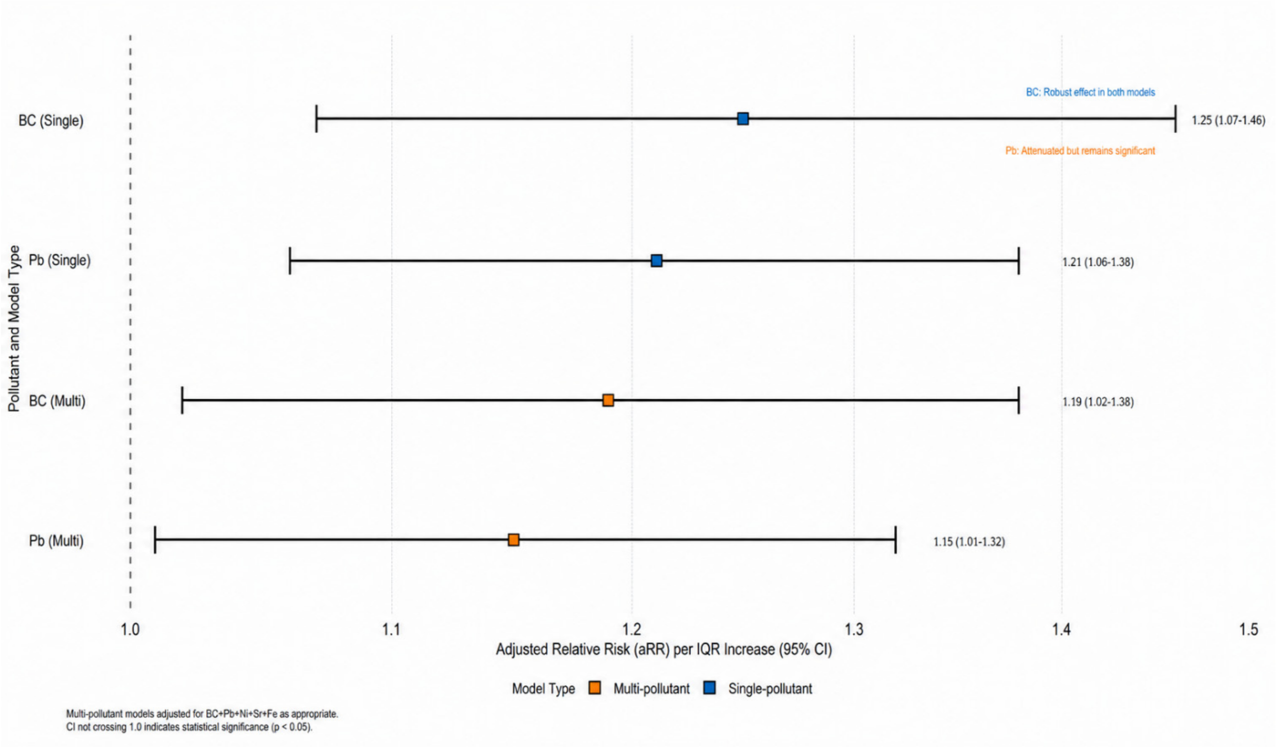

Figure S8 independent effects of BC and Pb in single and multi-pollutant model for CVD including HTN outcome (Inpatient)

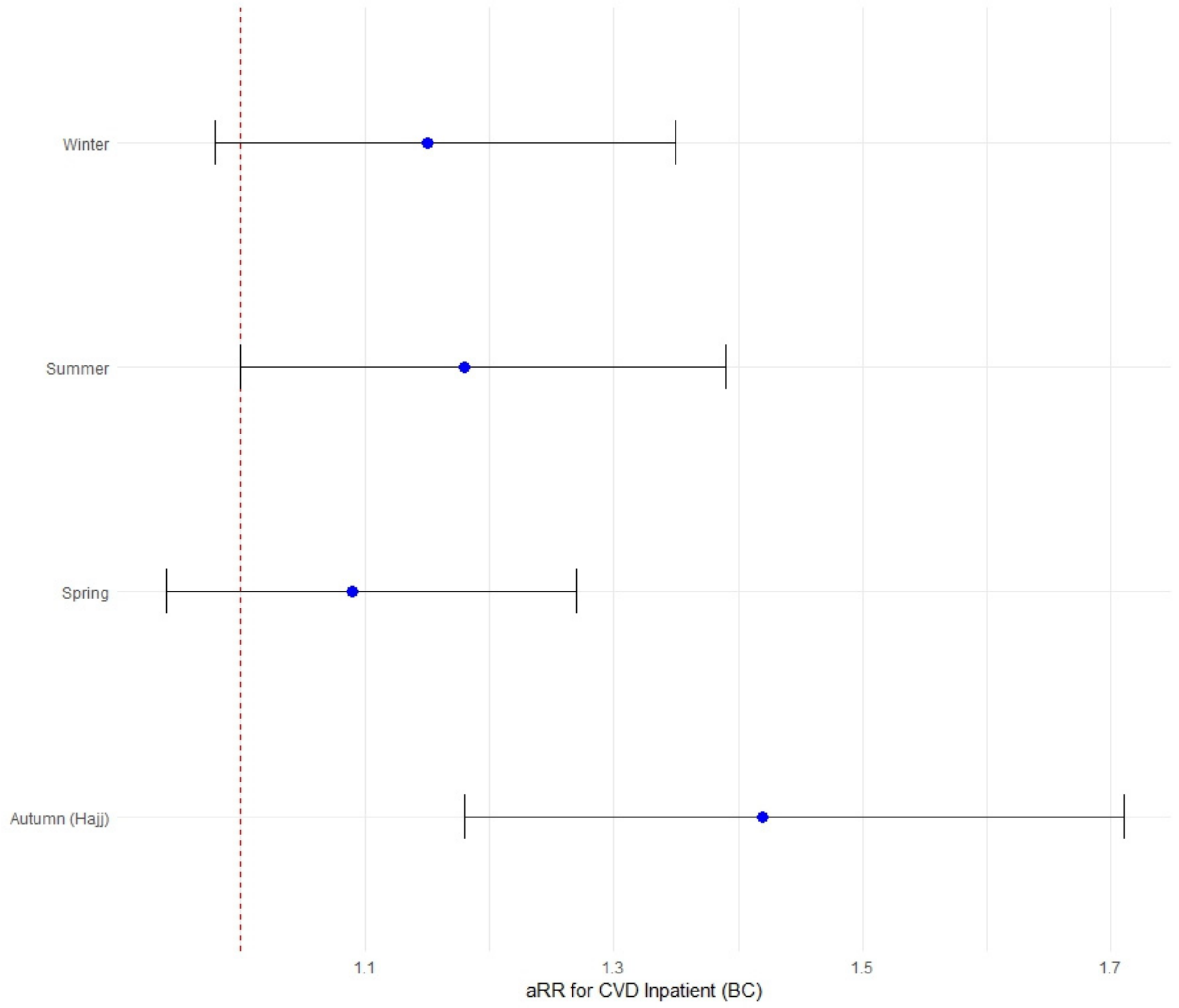

Figure S9 Seasonal forest plot for BC and CVD inpatient admissions

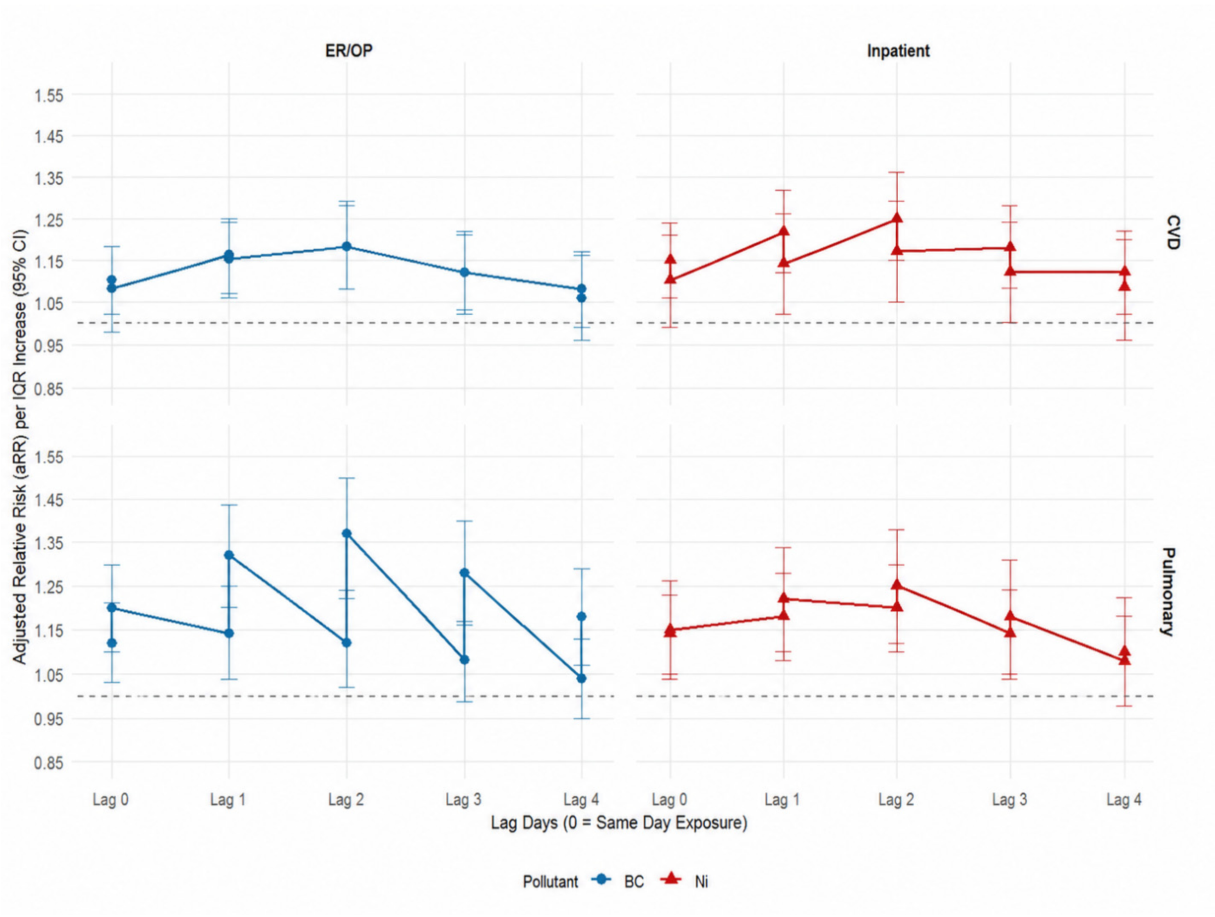

Figure S10 line graph for the effects of BC and Ni Inpatients and ER/OP at different lag days (lag0–lag4)
